# Pharmacogenetics of opioid medications for relief of labor pain and post-cesarean pain: a systematic review and meta-analysis

**DOI:** 10.1007/s00228-024-03798-z

**Published:** 2025-01-07

**Authors:** Martina Giacon, Sarah Cargnin, Maria Talmon, Salvatore Terrazzino

**Affiliations:** 1 Department of Pharmaceutical Sciences, University of Piemonte Orientale “A. Avogadro”, Largo Donegani 2, 28100 Novara, Italy; 2 Department of Health Sciences, Università del Piemonte Orientale (UPO), 28100 Novara, Italy

**Keywords:** Labor pain, Post-cesarean pain, Opioids, Gene polymorphisms, Meta-analysis

## Abstract

**Objective:**

Several studies have attempted to identify genetic determinants of clinical response to opioids administered during labor or after cesarean section. However, their results were often contrasting. A systematic review and meta-analysis was conducted to quantitatively assess the association between gene polymorphisms and clinical outcomes of opioid administration in the treatment of labor pain and post-cesarean pain.

**Methods:**

A comprehensive search was performed up to December 2023 using PubMed, Web of Knowledge, Cochrane Library, and OpenGrey databases. The clinical endpoints of interest were pain score after opioid treatment, total opioid consumption, patient’s analgesic satisfaction, and incidence of opioid side effects. Random-effects meta-analyses were conducted when data were available in at least three studies.

**Results:**

Twenty-six studies enrolling 7765 patients were included in the systematic review. Overall, a total of 12 candidate polymorphic genes (OPRM1, COMT, CYP2D6, CYP3A4, ABCB1, ABCC3, UGT2B7, CGRP, OPRK1, OPRD1, KCNJ6, KCNJ9) were considered by the included studies, among which the most investigated variant was OPRM1 rs1799971. Overall pooled results indicated that individuals carrying the G allele of *OPRM1* rs1799971 required higher opioid doses for pain management in comparison to rs1799971 AA subjects (standardized mean difference: 0.26; 95% CI: 0.09–0.44; *P* = 0.003). Such an association was confirmed in the subgroups of patients with labor pain and post-cesarean pain.

**Conclusion:**

The present meta-analysis provides strong evidence of an association between *OPRM1* rs1799971 and opioid dose requirement for relief of labor pain or post-cesarean pain. However, given the insufficient evidence for other polymorphic gene variants, large studies are still needed to investigate the impact of genetic variability on the efficacy and safety of opioid medications for relief of labor pain and post-cesarean pain (INPLASY Registration No. 202410040).

**Supplementary Information:**

The online version contains supplementary material available at 10.1007/s00228-024-03798-z.

## Introduction

Labor pain and delivery pain are among the worst pains that women experience in their lives [1]. Although not life-threatening, labor pain has relevant short- and long-term consequences for the well-being of mother and fetus. Poorly controlled labor pain can exacerbate maternal stress and anxiety, prolong labor, and lead to fetal metabolic acidosis [2, 3]. A significant proportion of postpartum women experience uterine involution pain, perineal pain, and incisional pain following cesarean section, which evolve into chronic pain in 6.1 to 11.5% of women [4, 5]. Notably, patients who do not receive adequate analgesia before and after labor are at higher risk of postpartum depression and post-traumatic stress syndrome [6, 7]. Therefore, the provision of analgesics to mitigate such harmful effects is becoming increasingly common, especially in high-income countries [8].

Opioids are drugs often used for the management of labor and post-cesarean pain [9–11]. However, patient response to opioids is highly variable in terms of pain relief, occurrence, and severity of side effects [12]. There are several factors that can contribute to the inter-individual variability of opioid response. These include clinical factors, such as age, parity, stage of labor, ethnicity [13, 14], and genetic factors [15]. Among the polymorphic variants of genes involved in opioid pharmacokinetics (e.g., *CYP2D6*, *CYP3A4*, *ABCB1*, *COMT*) or pharmacodynamics (e.g., *OPRM1*), much interest has been focused on *OPRM1* rs1799971 (A118G), a functional single nucleotide polymorphism (SNP) in the gene encoding the mu opioid receptor (MOR). This SNP consists of an Asn40Asp amino substitution in the extracellular N-terminus of the receptor which leads to reduced MOR density on neuronal membranes and altered MOR-mediated responses [16]. Some studies have shown that carriers of *OPRM1* rs1799971 G allele require higher opioid doses to achieve pain relief during labor [17] or after a cesarean section [18–20], when compared to homozygotes for the rs1799971 A allele. Conversely, some other studies suggest that the *OPRM1* A118G polymorphism does not affect the opioid dose requirement in both clinical settings [21–23]. Given the conflicting results of previous studies and the increasing number of publications in this field, we herein conducted a systematic review of published studies aimed at quantitatively summarizing the evidence on the association between gene polymorphisms and clinical outcomes of opioid medications in the context of labor and post-cesarean pain management.

## Methods

This meta-analysis follows the Meta-analysis Of Observational Studies in Epidemiology (MOOSE) guidelines. The MOOSE checklist is available in the Supplementary Materials (Supplementary Table 1).

### Literature search and inclusion/exclusion criteria

The protocol was set a priori to the literature search and registered on the International Platform of Registered Systematic Review and Meta-analysis Protocols (INPLASY) (Registration No. 202410040, doi:https://doi.org/10.37766/inplasy2024.1.0040). A comprehensive search of electronic databases (PubMed, Web of Knowledge, Cochrane Library, and OpenGrey) was conducted up to November19^th^, 2024, to identify potentially eligible studies. A Boolean combination of the following keywords was used: (opioid* OR opiate* OR analges*) AND (polymorphism* OR SNP OR SNPs OR variant* OR pharmacogenetic* OR pharmacogenomic*) AND (labor OR labour OR birth OR childbirth OR afterbirth OR “after-birth” OR “post-childbirth” OR prebirth OR “pre-birth” OR “near-birth” OR delivery OR “post-delivery” OR postdelivery OR “pre-delivery” OR predelivery OR postpartum OR “post-partum” OR antepartum OR “ante-partum” OR intrapartum OR “intra-partum” OR peripartum OR “peri-partum” OR partum OR natal OR prenatal OR “pre-natal” OR postnatal OR “post-natal” OR neonatal OR “neo-natal” OR perinatal OR “peri-natal” OR antenatal OR “ante-natal” OR intranatal OR “intra-natal”). To be included, studies had to meet the following eligibility criteria: (1) observational studies or randomized clinical trials including women treated with opioids by any route of administration for relief of labor pain or post-cesarean pain; (2) studies evaluating the association of any gene polymorphism with at least one of the following outcomes: (a) pain score after opioid administration based on any patient-reported scale; (b) total opioid consumption; (c) 50% effective opioid dose (ED50); (d) analgesic satisfaction based on any patient-reported scale; (3) studies with sufficient data to calculate the above-mentioned outcomes (pain score, total opioid consumption, ED50, and analgesic satisfaction) as mean ± standard deviation; (4) incidence of any specific adverse effect of opioid therapy. The following studies were excluded from systematic review: (1) not human studies; (2) studies not related to the research topics; (3) reports, case series, meeting abstract, editorials, letters to the editor, review articles, and meta-analyses; (4) studies not evaluating the association of gene polymorphisms with at least one of the outcomes of interest; (5) non-English publications, due to limited translation resources.

All potentially relevant studies identified in the first screening step were then read in full to check whether they were eligible for inclusion. A manual review of primary and review article references was also performed to identify additional relevant studies that had been overlooked in the initial electronic search. In cases where data for a given clinical outcome could not be extracted from an eligible study, the missing data were requested by email to the corresponding author of the study. The study was excluded from the systematic review or meta-analysis if the corresponding author did not respond to the email or did not provide the data requested for the effect size calculation. Two investigators (M.G. and S.C) independently screened the papers to minimize the risk of excluding relevant records and a third investigator (S.T.) resolved any discrepancies or conflicts. The citations located and those excluded, including justification, have been listed in an Excel file, which is available upon request.

### Data extraction

The following information was recorded for each included study: first author’s last name, year of publication, study location, patient ethnicity, type of pain (i.e., labor pain or post-cesarean pain), time of pain assessment, opioid administered and route of administration, total number of opioid-treated patients, reported outcome of interest, scale used for pain score assessment, gene variant investigated, and the genotyping method used. As for OPRM1 rs1799971, which was found the most investigated SNP, the minor allele frequency (MAF) and the *P*-value of Hardy Weinberg Equilibrium (HWE) test were also calculated from each study when genotype distribution was reported as three separate groups (AA, AG, GG), by an online HWE calculator (https://www.had2know.org/academics/hardy-weinberg-equilibrium-calculator-2-alleles.html). Means and standard deviations for continuous outcomes (pain score, total opioid consumption, ED50, and analgesic satisfaction), as well as incidence of adverse events following opioid therapy were extracted (or calculated) from each study for the three genotypes, when available as separate groups (homozygous major allele, heterozygous, and homozygous minor allele) or for combined groups as reported in the primary study. For continuous outcomes, regrouping heterozygous data to either homozygous group was calculated by combining means and standard deviations from each group, using an online tool available at https://www.statstodo.com/CombineMeansSDs.php. When data were reported as means with confidence intervals the standard deviation for each group was calculated by dividing the length of the confidence interval by 3.92 and then multiplying by the square root of the sample size, as reported by the Cochrane Handbook for Systematic Reviews of Interventions (https://training.cochrane.org/handbook/current/chapter-06#section-6-5-2-2). If the interquartile range (IQR, i.e., the difference between the third and first quartile values) was available, rather than standard deviation (SD), the standard deviation for each group was estimated with the formula proposed by the Cochrane handbook: SD = IQR/1.35 (https://training.cochrane.org/handbook/current/chapter-06#section-6-5-2–5). When median and range (minimum and maximum) or median, the first and the third quartile values were reported, the mean ± SD was estimated using an online tool available at https://www.math.hkbu.edu.hk/~tongt/papers/median2mean.html, which was also used to verify lack of data skewness. If the data were not skewed, mean and SD were used for calculation of the pooled estimate. In two cases [17, 24], the freely available software WebPlotDigitizer (version 4.6, https://automeris.io/WebPlotDigitizer/) was used to extract data from graphs. The data were independently extracted from eligible papers by two researchers (M.G. and S.T.), who subsequently cross-checked the data and resolved discrepancies.

### Assessment of study quality

Two reviewers (M.G. and S.C.) independently assessed the quality of the included studies using the Q-Genie tool and any disagreement was resolved by consensus or by a third reviewer (S.T.). The Q-Genie tool has been validated for assessing the quality of genetic association studies and is a Likert-type scale consisting of eleven questions (item 1, rationale for study; item 2, selection and definition of outcome of interest; item 3, selection and comparability of comparison groups; item 4, technical classification of the exposure; item 5, non-technical classification of the exposure; item 6, other sources of bias; item 7, sample size and power; item 8, a priori planning of analyses; item 9, statistical methods and control for confounding; item 10, testing of assumptions and inferences for genetic analyses; and item 11, appropriateness of inferences drawn from results), with a maximum score of 7 and a minimum score of 1 for each question [25]. For studies with a control group, a score < 35 indicates poor quality, 35–45 moderate quality, and > 45 good quality. For studies without a control group, a score < 32 indicates low quality, 32–40 moderate quality, and > 40 good quality.

### Statistical analysis

The mean difference (MD) and its 95% confidence intervals were used as summary statistics in meta-analyses of continuous variables if the outcome measures were on the same scale in all studies, otherwise the standard mean difference (SMD) and its 95% confidence intervals were derived for each study and used for pooling the results. If repeated time observations were reported in a single study, data with a normal distribution at the longest time point were used to determine the effect size of each study. With regard to the pooled risk of a given adverse effect, the odds ratio (OR) with 95% confidence intervals was calculated for each study and used as the summary effect size. Studies, irrespectively from the effect size, were pooled with the random-effect model, which assumes that the true effect size may differ from study to study due to differences (heterogeneity) among studies [26]. The inverse variance weighted average statistical method was used for all comparisons of MD or SMD, while the Mantel–Haenszel statistical method was used for pooling ORs. Heterogeneity between studies was estimated using the chi-squared-based Cochran’s *Q* test and its statistical significance was set at* P* < 0.10. Between-study heterogeneity was also estimated by the I^2^ statistic, with an I^2^ value ranging from 0 to 25% indicating insignificant heterogeneity, 26–50% implying low heterogeneity, 51–75% representing medium heterogeneity, and 76–100% indicating high heterogeneity. Meta-analyses were performed for each gene polymorphism and outcome of interest when data were available from at least three studies, and subgroup analyses were performed based on the type of pain (i.e., labor pain or post-cesarean pain). In addition, leave-one-out sensitive meta- analyses were performed to assess the contribution of each study to the pooled estimate by excluding individual results one at a time and recalculating the pooled OR estimates for the remaining results. All meta-analyses were conducted using the Review Manager (RevMan) 5.4.1 software and the statistically significant threshold for pooled analyses was set at *P* < 0.05. The presence of publication bias was assessed graphically by drawing funnel plots and statistically by the Egger’s test if at least ten studies were present in the pooled analysis. If there was statistical evidence of asymmetry in the funnel plot (Egger’s *P*-value < 0.10), the “trim-and-fill” method was used to adjust the overall effect estimate. The ProMeta version 2 software (INTERNOVI di Scarpellini Daniele s.a.s., Cesena FO, Italy) was used for performing leave-one-out meta-analyses and assessment of publication bias and trim-and-fill analysis.

## Results

### Literature search

**Fig. 1:**
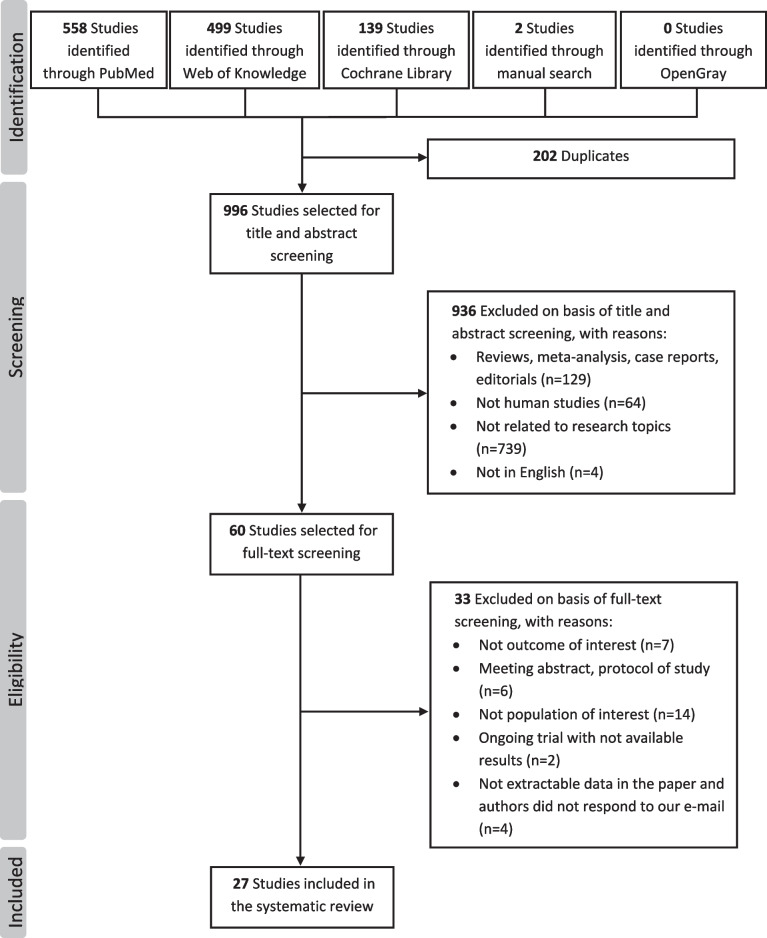
Flowchart of literature search and selection process of eligible studies

A total of 1198 studies were identified from databases (PubMed, *n* = 558; Web of Knowledge, *n* = 499; Cochrane Library, *n* = 139; OpenGray, *n* = 0) and manual literature search (*n* = 2). After removing 202 duplicates and 969 reports not fulfilling inclusion and exclusion criteria, 27 studies, all observational studies in study design, published between 2008 and 2024 were finally included in the systematic review [17–24, 27–45]. A detailed flowchart of the study selection process is shown in Fig. 1.

### General characteristics and quality of included studies

The summarized characteristics of the included studies are shown in Table 1. Twelve studies included Asian patients from China and Singapore [17, 18, 20, 24, 28, 29, 37, 39, 41–45]; 9 studies enrolled multi-ethnic patients from the USA, Canada, Switzerland, Israel, and Singapore [19, 21–23, 27, 31, 33, 34, 36]; 4 studies included Caucasian patients from Europe [30, 32, 35, 38]; and 1 study enrolled patients from the Middle East [40]. Seventeen studies recruited patients with post-cesarean pain [18–20, 22–24, 28–31, 35–39, 41, 42], 9 studies enrolled patients with labor pain [17, 27, 32–34, 40, 43–45], and 1 study included patients with both types of pain [21]. In terms of prescribed opioids, morphine was administered in 7 studies [18, 19, 28, 29, 35, 38, 41], sufentanil in 8 studies [17, 24, 30, 32, 37, 43–45], fentanyl in 6 [20, 27, 33, 34, 39, 40], codeine in 2 [23, 31], hydrocodone and hydromorphone in 2 [22, 36], fentanyl with morphine in 1 [21], and sufentanil with tramadol in 1 study [42]. Overall, a total of 12 candidate polymorphic genes were analyzed (*OPRM1*, *COMT*, *CYP2D6*, *CYP3A4*, *ABCB1*, *ABCC3*, *UGT2B7*, *CGRP*, *OPRK1*, *OPRD1*, *KCNJ6*, *KCNJ9*), among which *OPRM1* rs1799971 was investigated in 17 studies [17–23, 27, 29, 30, 32–34, 37, 38, 40, 42]. As for the outcome investigated, the majority of the included studies assessed pain score after opioid administration (*n* = 20), followed by total opioid consumption (*n* = 15), analgesic satisfaction (*n* = 4), and half-maximal effective opioid concentration (EC50, *n* = 2). In addition, pruritus, nausea, and vomiting were the most frequent types of adverse effects investigated. The Q-Genie tool indicated that 22 studies were of good quality, 4 were of moderate quality, and 1 study was of low quality, indicating an overall good methodological quality of the considered studies. The results of the quality assessment for each study are presented in Supplementary Table 2. Meta-analyses with at least three studies were conducted for OPRM1 rs1799971, ABCB1 rs1128503, COMT rs4680, CYP3A4 rs2242480, and genetically predicted CYP2D6 phenotype. Results of quantitative data synthesis for OPRM1 rs1799971 are presented below, while results for the other genetic variants are described and presented in the Supplementary Material.

**Table 1:** Main characteristics of studies included in the systematic review

Author, year [Ref]	Country (ethnicity)	Type of pain	Opioid (adm. route)	Total N subjects (N genotyped)	Gene variant (MAF of rs1799971/P_HWE_) (genotyping method)	Analgesic outcome of interest	Side effects	*Time of outcomes assessment*
Landau R, 2008 [27]	Switzerland (Mixed)	L	Fentanyl (intrathecal)	224 (158)	*OPRM1* rs1799971 (0.18/0.10) (pyrosequencing)	① (VRS) ③ (μg)	-	Over first 60 min after opioid administration
Sia AT, 2008 [18]	Singapore (Chinese)	P	Morphine (intravenous)	631 (588)	*OPRM1* rs1799971 (0.34/0.007) (TaqMan real-time PCR)	① (VAS) ② (mg)	Vomit, nausea, pruritus	Over first 24 h post-surgery
Tan E, 2009 [19]	Singapore (Mixed)	P	Morphine (intravenous)	1066 (994)	*OPRM1* rs1799971 (0.39/0.012) (TaqMan real-time PCR)	① (VAS) ② (mg)	-	Over first 24 h post-surgery
Sia AT, 2010 [28]	Singapore (Chinese)	P	Morphine (intravenous)	631 (620)	*ABCB1* rs1128503, rs2032582, rs1045642 (PCR–RFLP)	① (VAS) ② (mg)	CPSP	① and ② over first 24 h post-surgery; side effect at 3 months post-surgery
Tsai FF, 2010 [29]	China (Chinese)	P	Morphine (epidural)	217 (212)	*OPRM1* rs1799971 (0.33/0.43) (light cycler real-time PCR)	-	Pruritus	At 24 h post-surgery
Wong CA, 2010 [21]	USA (Mixed)	L /P	Fentanyl and morphine (intrathecal)	L: 210 (190) P: 105 (103)	*OPRM1* rs1799971 (L: 0.15/ < 0.001; P: 0.14/0.36) (pyrosequencing)	① (VAS) ② (μg/mg) ④ (VAS)	Vomit, nausea, pruritus	During labor: ① at 1st analgesia request, 10 min after opioid administration and at 2nd analgesia request; ② during labor; ④ at 1 h after delivery; side effects at 2nd request for analgesia Post-operative period: ① at 1st rescue analgesia request; ② over 72 h post-surgery; ④ at 24 h post-surgery; side effects over first 24 h post-surgery
De Capraris A, 2011 [30]	Italy (Caucasian)	P	Sufentanil (epidural)	80 (22)	*OPRM1* rs1799971 (0.11/0.55) (Sanger sequencing)	① (VAS)	-	At 6, 24, 48 h post-surgery
VanderVaarrt S, 2011 [31]	Canada (Mixed)	P	Codeine (oral)	80 (45)	*CYP2D6* *2, *3, *4, *5, *6, *7, *8, *9, *10, *12, *14, *17, *29, *41, *XN (multiplexed microarray assay)	① (VAS) ② (mg/kg)	-	At day 2 and 3 post-surgery
Camorcia M, 2012 [32]	Italy (Caucasian)	L	Sufentanil (epidural)	77 (57)	*OPRM1* rs1799971 (0.22/0.18) (pyrosequencing)	③ (μg)	-	Over first 30 min after opioid administration
Landau R, 2013 [33]	USA (Mixed)	L	Fentanyl (intravenous)	OPRM1: 106 (98) COMT: 106 (100)	*OPRM1* rs1799971 (0.22/0.97), *COMT* rs4680 (pyrosequencing)	④ (NVPS)	-	At 15 min after opioid administration
Boswell MV, 2013 [22]	USA (Mixed)	P	Hydrocodone and hydromorphone (oral)	158 (158)	*OPRM1* rs1799971 (0.10/0.01) (bead-based multiplex assay)	① (VAS) ② (mg)	Vomit, nausea, pruritus, confusion, weakness, constipation, dizziness, dry mouth, loss of appetite, respiratory depression, sleep disturbance, sonnolence, sweating,	At 72 h post-surgery
Ginosar Y, 2013 [34]	USA/Israel (Mixed)	L	Fentanyl (epidural)	125 (125)	*OPRM1* rs1799971 (0.15/0.85) (PCR–RFLP)	① (VAPS)	-	At request for analgesia
Quinta R, 2014 [35]	Portugal (Caucasian)	P	Morphine (NR)	55 (55)	*CYP2D6* rs1135840, rs35742686, rs3892097, rs5030655 (TaqMan real-time PCR)	-	Pruritus	Over first 12 h post- surgery
Stauble ME, 2014 [36]	USA (Mixed)	P	Hydrocodone and hydromorphone (oral)	156 (156)	*CYP2D6* rsIDs NR (xTAG Mutation Detection assay)	① (VAS) ② (mg)	-	At day 3 after surgery
Baber M, 2015 [23]	Canada (Mixed)	P	Codeine (oral)	255 (98)	*OPRM1* rs1799971 (0.21/0.13), *COMT* rs4633, rs4818, rs4680, *CYP2D6* rsIDs NR, *ABCB1* rs1128503, rs2032582, rs1045642, *UGT2B7* rs7439366 (TaqMan real-time PCR)	① (VAS) ② (mg/kg)	-	Over first 48 h post-surgery
Xu GH, 2015 [37]	China (Chinese)	P	Sufentanil (epidural)	180 (161)	*OPRM1* rs1799971 (0.36/0.22) (Sanger sequencing)	① (VAS) ② (mL) ④ (VRS)	Nausea, pruritus	① at 6, 12, 24 h post-surgery; ② ④ and side effects over first 24 h post-surgery
Pettini E, 2018 [38]	Italy (Caucasian)	P	Morphine (intrathecal)	63 (63)	*OPRM1* rs1799971 (0.15/0.67) (TaqMan real-time PCR)	-	Pruritus, PONV	At 24 h and 48 h post-surgery
Xie W, 2018 [39]	China (Chinese)	P	Fentanyl (epidural)	548 (521)	*CGRP* 4218 T/C (PCR–RFLP)	① (VAS) ② (μg/kg)	Vomit, nausea, pruritus	Over first 24 h post-surgery
Lv J, 2018 [24]	China (Chinese)	P	Sufentanil (intravenous)	208 (208)	*CYP3A4* rs2242480 (PCR–RFLP)	① (NRS) ② (μg)	-	At 8 h, 24 h, and 48 h post-surgery
Zhang J, 2018 [20]	China (Chinese)	P	Fentanyl (epidural)	240 (240)	*OPRM1* rs1799971 (0.35/0.72), *ABCB1* rs1128503, *CYP3A4* rs2242480 (Sanger sequencing)	① (VAS) ② (μg/kg)	Vomit, nausea, dizziness	① at 12 h and 24 h post-surgery; ② at 24 h and 48 h post-surgery; side effects at a not specified time
Zgheib NK, 2018 [40]	Lebanon (Middle Eastern)	L	Fentanyl (epidural)	250 (220)	*OPRM1* rs1799971 (0.12/0.06), *OPRM1* rs9479757 *OPRM1* rs2075572 (TaqMan real-time PCR)	① (VAS) ④ (VAS)	Vomit, nausea, pruritus	① at 1st request for analgesia, at 10, 20, and 30 min after opioid administration and at the 2nd request for analgesia; ④ over 1st 2 h after delivery; side effects at a not specified time
Kung CC, 2018 [41]	China (Chinese)	P	Morphine (epidural)	217 (217)	*CYP3A4* rs2242480, rs28371759, *CYP2D6* rs3892097, rs28365063, *UGT2B7* rs7439366, rs7439152, *ABCB1* rs1045642, *ABCC3* rs2277624, *OPRK1* rs1051660, *OPRD1* rs1042114, *KCNJ6* rs2070995, *KCNJ9* rs2737703 (TaqMan real-time PCR)	-	Pruritus	Over first 24 h post-surgery
Wang L, 2019 [42]	China (Chinese)	P	Sufentanil and tramadol (intravenous)	830 (266)	*OPRM1* rs1799971 (0.30/0.020), *COMT* rs4680 (TaqMan real-time PCR)	① (VAS) ② (mL)	CPSP	① at 24 h and 48 h post-surgery; ② at 12 h, 24 h and 48 h post-surgery; side effect at 3 months post-surgery
Xiaohong Y, 2020 [43]	China (Chinese)	L	Sufentanil (epidural)	97 (97)	*COMT* rs4680 (light cycler real-time PCR)	② (mL)	-	NR
Chen Y, 2023 [17]	China (Chinese)	L	Sufentanil (epidural)	356 (240)	*OPRM1* rs1799971 (0.36/0.87), *COMT* rs4680 (Sanger sequencing)	② (μg)	PONV, pruritus, urine retention, fever, tachycardia, hypotension, respiratory inhibition,	② during labor; side effects over first 20 min after opioid administration
Shu X, 2024 [44]	China (Chinese)	L	Sufentanil (epidural)	600 (573)	*CYP3A4* rs2242480 (TaqMan real-time PCR)	① (VAS) ② (mg)	Nausea, vomit, pruritus, hypotension, dizziness, uroschesis	② during labor; ① and side effects at 1 h and 3 h after opioid administration
Li W, 2024 [45]	China (Chinese)	L	Sufentanil (epidural)	239 (239)	*ABCB1* rs1128503*, ABCB1* rs1045642 (PCR–RFLP)	① (VAS)	Dizziness, nausea, urinary retention, constipation, pruritus	① at 1 h and 2 h after opioid administration; side effects after opioid administration

①, pain score after opioid administration based on any patient-reported scale; ②, total opioid consumption; ③, 50% effective opioid dose (ED50); ④, analgesic satisfaction based on any patient reported scale; pain scale or unit of measure used is indicated in the brackets. Abbreviations: *Adm*., administration; *CPSP*, chronic postsurgical pain; *L*, labor pain; *MAF*, minor allele frequency; *NR*, not reported; *NRS*, Numerical Rating Scale; *NVPS*, Numerical Verbal Pain score; *P*, post-cesarean pain; *P*_*HWE*_, P-value of Hardy Weinberg Equilibrium; *PONV*, postoperative nausea and vomiting; *RFLP*, restriction fragment length polymorphism; *USA*, United States of America; *VAS*, Visual Analog Score; *VAPS*, Visual Analogue Pain scale; *VRS*, Verbal Rating Scale

### Association of *OPRM1* rs1799971 with pain score after opioid treatment

**Fig. 2:**
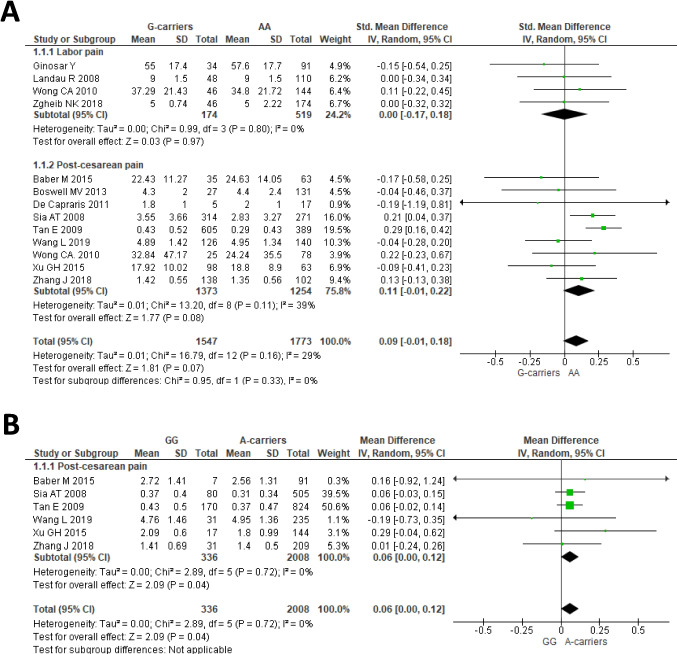
Forest plots of the standardized mean differences of pain score after opioid treatment for relief of labor pain and post-cesarean pain, for the dominant model (GG or AG vs AA) (**A**) or for the recessive model (GG vs AG or AA) (**B**) of *OPRM1* rs1799971. Note that the diamond symbol in B is shown twice to emphasize the lack of studies for the subgroup of patients with labor pain

Thirteen cohorts from 12 studies, enrolling a total of 3320 patients, were available for the meta-analysis of the dominant model of *OPRM1* rs1799971 (GG or AG vs AA) and association with pain score after opioid treatment [18–23, 27, 30, 34, 37, 40, 42]. The pooled results showed no association with pain score under the dominant model of rs1799971 (Fig. 2A), both in the overall analysis, as well as in the subgroup of subjects with pain after cesarean section or patients with labor pain. In the overall analysis, a significant funnel plot asymmetry was detected according to the Egger’s test (*P* = 0.001) and the subsequent trim-and-fill analysis identified six missing studies on the right side of the funnel plot (Supplementary Fig. 1). The adjusted trim-and-fill effect size was significant (SMD = 0.18, 95% CI 0.08–0.28. *P* = 0.001), indicating that the observed effect size was influenced by missing studies.

Six studies, including a total of 2344 patients, were available for the meta-analysis between the recessive model of *OPRM1* rs1799971(GG vs AG or AA) and pain score after opioid treatment [18–20, 23, 37, 42]. The Cochran’s *Q* test revealed absence of between-study heterogeneity under the recessive model (I^2^ = 0%, *P* = 0.72). A significant difference of pain score was detected in the pooled analysis (Fig. 2B), which included patients with post-cesarean pain only (MD: 0.06; 95% CI: 0.00–0.12; *P* = 0.04).

### Association of *OPRM1* rs1799971 with total opioid consumption

**Fig. 3:**
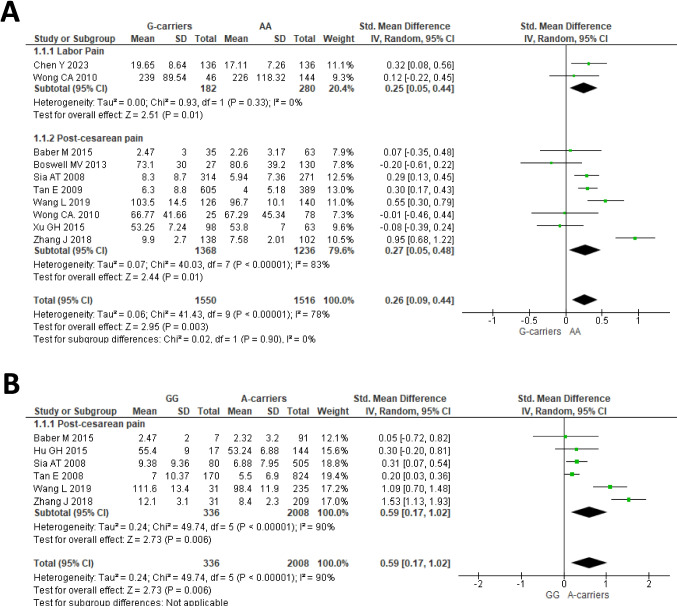
Forest plots of the standardized mean differences of total opioid consumption after opioid treatment for relief of labor pain and post-cesarean pain, for the dominant model (GG or AG vs AA) (**A**) or for the recessive model (GG vs AG or AA) (**B**) of *OPRM1* rs1799971. Note that the diamond symbol in B is shown twice to emphasize the lack of studies for the subgroup of patients with labor pain

Ten cohorts from 9 studies [17–23, 37, 42] were included in the meta-analysis of the dominant model of *OPRM1* rs1799971 and association with total opioid consumption. In the overall analysis (Fig. 3A), the pooled results indicated that individuals carrying the G allele (AG or GG) of rs1799971 required higher opioid doses for pain management compared to AA homozygous subjects (SMD: 0.26; 95% CI: 0.09–0.44; *P* = 0.003). The results were stable in sensitivity analysis (Supplementary Fig. 2A) with pooled SMD values ranging between 0.26 (95%CI: 0.09–0.44, *P* = 0.003) and 0.46 (95%CI: 0.26–0.67, *P* < 0.0001), and no publication bias was detected in the funnel plot (Egger’s test *P*-value = 0.431, Supplementary Fig. 3). In addition (Fig. 3A), a significant effect of the dominant model of rs1799971 on total opioid consumption was detected also in the subgroups of subjects with labor pain (SMD: 0.25; 95% CI: 0.05–0.44; *P* = 0.01) and post-cesarean pain (SMD: 0.27; 95% CI: 0.05–0.48; *P* = 0.01).

Six studies including patients with post-cesarean pain only [18–20, 23, 37, 42] were available for the association between the recessive model of *OPRM1* rs1799971 and total opioid consumption (Fig. 3B). A significant difference was observed among carriers of rs1799971 GG genotype in comparison to carriers of the A-allele in terms of opioid dose requirement for pain management of post-cesarean pain (SMD: 0.59; 95% CI: 0.17–1.02; *P* = 0.006, Fig. 3B). The results were stable in sensitivity analysis (Supplementary Fig. 2B), with pooled SMD values ranging between 0.41 (95%CI: 0.09–0.72, *P* = 0.011) and 0.67 (95%CI: 0.20–1.14, *P* = 0.005).

### Association of *OPRM1* rs1799971 with patient’s analgesic satisfaction and risk of opioid side effects

**Fig. 4:**
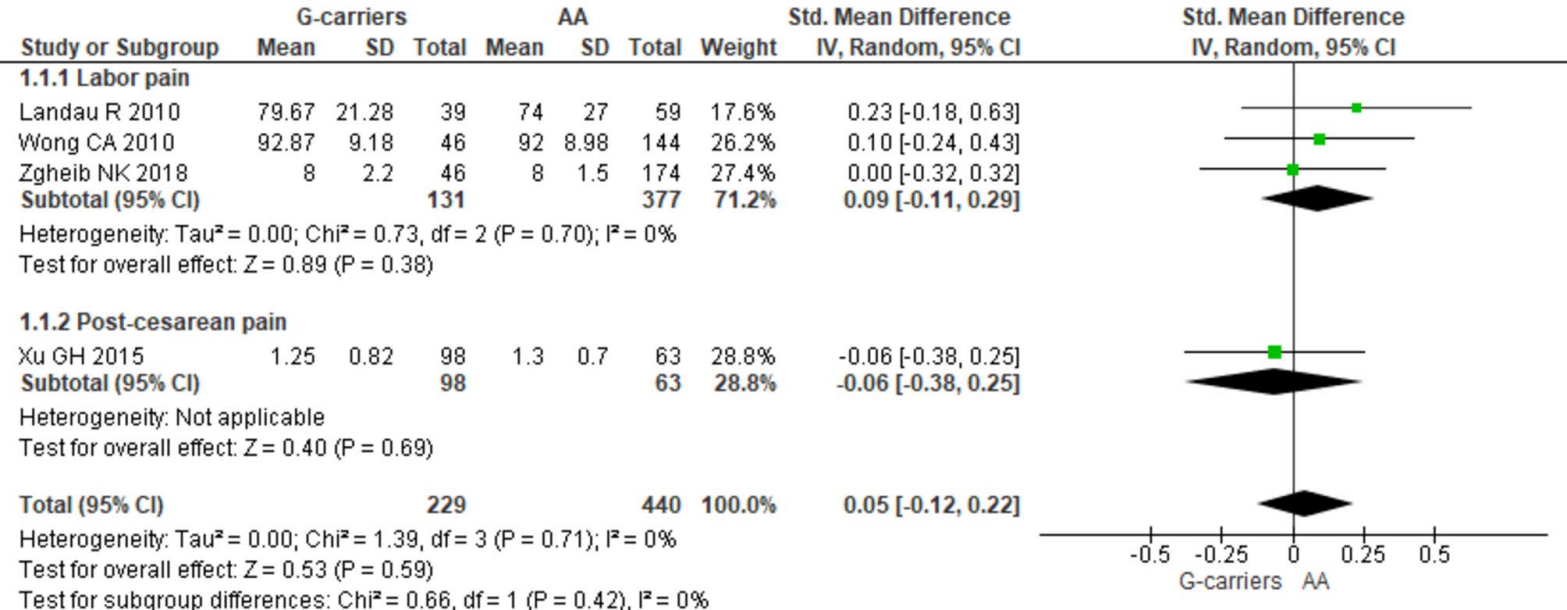
Forest plot of the standardized mean difference of patient’s analgesic satisfaction after opioid treatment for relief of labor pain and post-cesarean pain, for the dominant model of *OPRM1* rs1799971 (GG or AG vs AA)

Four studies [21, 27, 37, 40] involving 669 subjects were available for the effect of the dominant model of *OPRM1* rs1799971 (GG or AG vs AA) on patient’s analgesic satisfaction (Fig. 4). No significant differences were detected in the pooled results between carriers of the rs1799971 G allele in comparison to carriers of the rs1799971 AA genotype, in both the overall and subgroup analyses (Fig. 4). As regard to the association of *OPRM1* rs1799971 with the risk of opioid side effects, meta-analyses with at least three studies were available for pruritus (*N*_studies_ = 9), nausea (*N*_studies_ = 7), and vomiting (*N*_studies_ = 5). No significant differences were found in the overall analyses or in the subgroups of patients under either the dominant or the recessive genetic model of rs1799971 (Supplementary Figs. 4, 5 and 6).

## Discussion

In the present study we conducted a systematic review and meta-analysis of the association between genetic polymorphisms and efficacy and safety of opioids administered for pain relief during labor or after cesarean section. The pooled results of good/moderate quality of the included studies highlight *OPRM1* rs1799971 as a determinant of opioid consumption for labor and post-cesarean pain relief, with carriers of the variant G allele requiring higher opioid doses compared to AA subjects. The robustness of this finding was proven by sensitivity meta-analysis that showed stability of the pooled effect estimate. In addition, the recessive model of rs1799971 was found associated with pain scores and total opioid consumption of patients with post-cesarean pain. Conversely, all overall meta-analyses conducted for *ABCB1* rs1128503, *COMT* rs4680, *CYP3A4* rs2242480, and genetically predicted *CYP2D6* phenotype revealed no significant association with the clinical endpoint investigated, which included pain score, total opioid consumption, patient’s analgesic satisfaction, and opioid side effects.

*OPRM1* rs1799971 (118 A > G) is a functional variant that has long been considered as one of the most promising genetic candidates for pain sensitivity and opioid analgesia [46]. *OPRM1* rs1799971 leads to a non-synonymous A > G substitution at position 118 of *OPRM1*, which exchanges an Asn residue at amino acid position 40 of the N-terminal extracellular region of the receptor for an Asp. In wild-type receptors, the Asn residue serves as a site for N-glycosylation, which is completely removed by the amino acid exchange for Asp [47]. The first molecular consequence discovered for rs1799971 was an approximately three times higher affinity of beta-endorphin for the variant MORs compared to the most common allelic form of the receptor. However, such evidence was not confirmed for exogenous opioids, such as fentanyl and morphine, for which the binding affinity to MOR resulted unaltered [48]. Subsequently, rs1799971 was reported to reduce the signaling efficiency of MOR by more than a third when induced by exogenous opioids [49] and to a decrease of MOR expression, with carriers of the G allele showing halved *OPRM1* mRNA levels in the brain compared to wild-type A carriers [50]. Interestingly, rs1799971 appears to affect MOR expression in the brain via a genetic-epigenetic interaction conferred by the minor 118G allele: rs1799971 replaces the CA nucleotides at position 117/118 with CG, leading to the introduction of a new CpG site that confers a higher methylation degree, which in turn impedes MOR upregulation in brain tissues [51]. Since opioid-mediated analgesia mainly relies on MOR density, carriers of the variant G-allele have been postulated to exhibit poor response to opioids. Nevertheless, to date, no standardized genotype-to-phenotype groupings have been proposed for *OPRM1* and none of the current guidelines recommends the *OPRM1* genotype-guided dosing of opioids in any pain setting [52].

Our pooled results showed that carriers of the variant rs1799971 G allele require higher opioid doses for achieving adequate pain relief compared to wild-type homozygous patients. These findings differ from a previous meta-analysis published in 2013 [53], which showed that women carrying the rs1799971 G allele require less fentanyl doses for labor pain compared with those with the AA genotype. However, such discrepant result can be ascribed to the smaller sample size of the previous meta-analysis (*N* = 460) compared to the one included in the present study (*N* = 3066). On the other hand, our results are in the same direction of association as compared to previous meta-analyses evaluating the relationship between rs1799971 and opioid consumption in patients with postsurgical pain [54, 55]. It is noteworthy that previous meta-analyses focusing on different types of postsurgical pain [54, 55] included very few studies on post-cesarean pain, which in turn may differ from other types of post-operative pain [56]. In addition, our pooled results showed a statistically significant association between the recessive model of rs1799971 and pain score after opioid treatment in patients with post-cesarean pain, with carriers of rs1799971GG genotype showing higher pain scores compared to carriers of the A allele. On the other hand, the present meta-analysis revealed no significant association between the dominant model of rs1799971 and pain score after opioid treatment. Nevertheless, the trim-and-fill-adjusted result was significant, suggesting that the pooled effect size may be underestimated due to possible missing studies. This latter result is in line with results of the above-mentioned meta-analyses conducted on patients with post-surgical pain, which reported significant higher postoperative pain scores after opioid treatment among carriers of the G allele of rs1799971 when compared to AA individuals [54, 55]. As regard to the association of *OPRM1* rs1799971 with opioid side effects, no association was found in overall analyses, as well in the subgroups of patients with labor pain and post-cesarean pain. Although sparse evidence exists on the association of rs1799971 with nausea and vomiting induced by opioids when administered for postoperative pain [57, 58], our findings are consistent with the results of a recent meta-analysis that showed no impact of rs1799971 on the risk of nausea or vomiting after opioid administration in patients with postoperative pain [54].

We acknowledge some limitations of the present study that should be considered when interpreting the results. First, the sample size of our meta-analyses was relatively small, especially when meta-analyses were conducted in the subgroup of patients with labor pain or when the role of SNPs other than rs1799971 was investigated. In addition, Hardy–Weinberg imbalance was found in some of the included studies, so we cannot rule out population stratification or selection bias in these studies. Therefore, the results of the present meta-analysis should be interpreted with caution. Despite this, only 1 (4%) of the included studies was rated of poor quality, 4 studies (12%) were of moderate quality, and 22 studies (81%) of good quality, suggesting an overall good methodological quality of the considered studies. Second, we attempted to conduct a comprehensive systematic review of all available published reports; however, corresponding authors of some eligible studies were unable to provide the data requested for the effect size calculation or did not respond to our email. In addition, 4 non-English reports were identified and excluded from the systematic review due to limited translation resources. This may explain, at least in part, the asymmetry detected in the funnel plot for the association of rs1799971 with pain score. Nevertheless, the trim-and-fill-adjusted result was significant, suggesting that the pooled effect size may have been underestimated. Third, the studies included in the present systematic review differ with respect to the type of pain (i.e., labor pain or post-cesarean pain). However, for each gene polymorphism and outcome of interest, subgroup analyses were performed based on the type of pain. Moreover, the included studies differ with respect to some other characteristics, such as type of administered opioid, route of administration, patients’ ethnicity, and genotyping method, which may have an impact on the effect size estimation. The opioids investigated in the studies included in this systematic review differ in their pharmacokinetic and pharmacodynamic properties. For example, morphine, codeine (which is converted to morphine), fentanyl, and sufentanil act as complete agonists with high selectivity for µ-opioid receptors (MORs), their primary site of action. Although hydrocodone has a relatively weak binding affinity for MORs, it is metabolized by CYP2D6 through demethylation at the 3-carbon position to hydromorphone, a metabolite with significantly higher MOR affinity. Tramadol, a 4-phenyl-piperidine derivative of codeine, also shows high selectivity for MORs, but acts as a partial agonist at these receptors. However, despite these differences all opioid analgesics share the common characteristic that they interact with opioid receptors to relieve pain and that they have certain side effects, although some of these are more common with one drug than another. Nevertheless, the limited number of studies included in each meta-analysis hampered the possibility to deeply investigate factors potentially causing between-study heterogeneity, which emerged in some of the meta-analyses conducted. Lastly, due to the lack of access to original data of primary studies, our meta-analyses are based on unadjusted estimates, so the pooled results might be confounded by relevant covariates, such as age, sex, comorbidities, and adjuvant multimodal analgesia administered to manage labor and post-cesarean pain. Therefore, a meta-analysis of individual participant data should be performed in the future to obtain pooled estimates for SNPs adjusted for confounding variables.

## Conclusions

The present systematic review and meta-analysis reviewed the relationship between genetic variants and efficacy and safety of opioids for pain relief during labor or after cesarean section. The pooled results highlight *OPRM1* rs1799971 as a genetic factor with potential clinical utility in predicting opioid dose requirement in such pain settings. If further corroborated by larger studies, this pharmacogenetic factor could potentially assist clinicians in optimizing maternal pain management during labor and after cesarean section. However, given the insufficient evidence for other polymorphic variants, there remains a need to investigate the impact of genetic variability on the efficacy and safety of opioid medications in the contexts of labor-and post-cesarean pain management.

## Supplementary Information

Below is the link to the electronic supplementary material.

Supplementary file1 (PDF 532 KB)

## Data Availability

Data is provided within the manuscript or supplementary information files.

## References

[1] Thomson G, Feeley C, Moran VH, Downe S, Oladapo OT (2019) Women’s experiences of pharmacological and non-pharmacological pain relief methods for labour and childbirth: a qualitative systematic review. Reprod Health 16:71 31146759 10.1186/s12978-019-0735-4 PMC6543627

[2] Reynolds F (2010) The effects of maternal labour analgesia on the fetus. Best Pract Res Clin Obstet Gynaecol 24:289–302 20005180 10.1016/j.bpobgyn.2009.11.003

[3] Reynolds F (2011) Labour analgesia and the baby: good news is no news. Int J Obstet Anesth 20:38–50 21146977 10.1016/j.ijoa.2010.08.004

[4] Mackenzie J, Murray E, Lusher J (2018) Women’s experiences of pregnancy related pelvic girdle pain: a systematic review. Midwifery 56:102–111 29096278 10.1016/j.midw.2017.10.011

[5] Lavand’homme P (2019) Postpartum chronic pain. Minerva Anestesiol 85:320–324 30394066 10.23736/S0375-9393.18.13060-4

[6] Andersen LB, Melvaer LB, Videbech P, Lamont RF, Joergensen JS (2012) Risk factors for developing post-traumatic stress disorder following childbirth: a systematic review. Acta Obstet Gynecol Scand 91:1261–1272 22670573 10.1111/j.1600-0412.2012.01476.x

[7] Lim G, Farrell LM, Facco FL, Gold MS, Wasan AD (2018) Labor analgesia as a predictor for reduced postpartum depression scores: a retrospective observational study. Anesth Analg 126:1598–1605 29239949 10.1213/ANE.0000000000002720 PMC5908733

[8] Seijmonsbergen-Schermers AE, van den Akker T, Rydahl E, Beeckman K, Bogaerts A, Binfa L et al (2020) Variations in use of childbirth interventions in 13 high-income countries: a multinational cross-sectional study. PLoS Med 17:e1003103 32442207 10.1371/journal.pmed.1003103 PMC7244098

[9] Zuarez-Easton S, Erez O, Zafran N, Carmeli J, Garmi G, Salim R (2023) Pharmacologic and nonpharmacologic options for pain relief during labor: an expert review. Am J Obstet Gynecol 228:S1246–S1259 37005099 10.1016/j.ajog.2023.03.003

[10] Pharmacologic stepwise multimodal approach for postpartum pain management (2021) ACOG Clinical Consensus No. 1. Obstet Gynecol 138:507–517 34412076 10.1097/AOG.0000000000004517

[11] Smith A, Laflamme E, Komanecky C (2021) Pain management in labor. Am Fam Physician 103:355–364 33719377

[12] Nanji JA, Carvalho B (2020) Pain management during labor and vaginal birth. Best Pract Res Clin Obstet Gynaecol 67:100–112 32265134 10.1016/j.bpobgyn.2020.03.002

[13] Wong CA (2012) The promise of pharmacogenetics in labor analgesia…tantalizing, but not there yet. Int J Obstet Anesth 21:105–108 22405670 10.1016/j.ijoa.2012.02.003

[14] Anim-Somuah M, Smyth RM, Cyna AM, Cuthbert A (2018) Epidural versus non-epidural or no analgesia for pain management in labour. Cochrane Database Syst Rev 5:CD000331 29781504 10.1002/14651858.CD000331.pub4 PMC6494646

[15] Magarbeh L, Gorbovskaya I, Le Foll B, Jhirad R, Müller DJ (2021) Reviewing pharmacogenetics to advance precision medicine for opioids. Biomed Pharmacother 142:112060 34523422 10.1016/j.biopha.2021.112060

[16] Huang P, Chen C, Mague SD, Blendy JA, Liu-Chen LY (2012) A common single nucleotide polymorphism A118G of the μ opioid receptor alters its N-glycosylation and protein stability. Biochem J 441:379–386 21864297 10.1042/BJ20111050 PMC3923516

[17] Chen Y, Chen Q, Cai C, Lin X, Yu W, Huang H et al (2023) Effect of OPRM1/COMT gene polymorphisms on sufentanil labor analgesia: a cohort study based on propensity score matching. Pharmacogenomics 24:675–684 37610885 10.2217/pgs-2023-0103

[18] Sia AT, Lim Y, Lim EC, Goh RW, Law HY, Landau R et al (2008) A118G single nucleotide polymorphism of human mu-opioid receptor gene influences pain perception and patient-controlled intravenous morphine consumption after intrathecal morphine for postcesarean analgesia. Anesthesiology 109:520–526 18719451 10.1097/ALN.0b013e318182af21

[19] Tan EC, Lim EC, Teo YY, Lim Y, Law HY, Sia AT (2009) Ethnicity and OPRM variant independently predict pain perception and patient-controlled analgesia usage for post-operative pain. Mol Pain 5:32 19545447 10.1186/1744-8069-5-32 PMC2709614

[20] Zhang J, Zhang L, Zhao X, Shen S, Luo X, Zhang Y (2018) Association between MDR1/CYP3A4/OPRM1 gene polymorphisms and the post-caesarean fentanyl analgesic effect on Chinese women. Gene 661:78–84 29601950 10.1016/j.gene.2018.03.081

[21] Wong CA, McCarthy RJ, Blouin J, Landau R (2010) Observational study of the effect of mu-opioid receptor genetic polymorphism on intrathecal opioid labor analgesia and post-cesarean delivery analgesia. Int J Obstet Anesth 19:246–253 20171873 10.1016/j.ijoa.2009.09.005

[22] Boswell MV, Stauble ME, Loyd GE, Langman L, Ramey-Hartung B, Baumgartner RN et al (2013) The role of hydromorphone and OPRM1 in postoperative pain relief with hydrocodone. Pain Physician 16:E227–E235 23703421

[23] Baber M, Chaudhry S, Kelly L, Ross C, Carleton B, Berger H et al (2015) The pharmacogenetics of codeine pain relief in the postpartum period. Pharmacogenomics J 15:430–435 25752520 10.1038/tpj.2015.3

[24] Lv J, Liu F, Feng N, Sun X, Tang J, Xie L (2018) CYP3A4 gene polymorphism is correlated with individual consumption of sufentanil. Acta Anaesthesiol Scand 62:1367–1373 29926893 10.1111/aas.13178

[25] Sohani ZN, Meyre D, de Souza RJ, Joseph PG, Gandhi M, Dennis BB et al (2015) Assessing the quality of published genetic association studies in meta-analyses: the quality of genetic studies (Q-Genie) tool. BMC Genet 16:50 25975208 10.1186/s12863-015-0211-2 PMC4431044

[26] Borenstein M, Hedges LV, Higgins JP, Rothstein HR (2010) A basic introduction to fixed-effect and random-effects models for meta-analysis. Res Synth Methods 1:97–111 26061376 10.1002/jrsm.12

[27] Landau R, Kern C, Columb MO, Smiley RM, Blouin JL (2008) Genetic variability of the mu-opioid receptor influences intrathecal fentanyl analgesia requirements in laboring women. Pain 139:5–14 18403122 10.1016/j.pain.2008.02.023 PMC2669083

[28] Sia AT, Sng BL, Lim EC, Law H, Tan EC (2010) The influence of ATP-binding cassette sub-family B member -1 (ABCB1) genetic polymorphisms on acute and chronic pain after intrathecal morphine for caesarean section: a prospective cohort study. Int J Obstet Anesth 19:254–260 20627697 10.1016/j.ijoa.2010.03.001

[29] Tsai FF, Fan SZ, Yang YM, Chien KL, Su YN, Chen LK (2010) Human opioid μ-receptor A118G polymorphism may protect against central pruritus by epidural morphine for post-cesarean analgesia. Acta Anaesthesiol Scand 54:1265–1269 21039348 10.1111/j.1399-6576.2010.02310.x

[30] De Capraris A, Cinnella G, Marolla A, Salatto P, Da Lima S, Vetuschi P et al (2011) Micro opioid receptor A118G polymorphism and post-operative pain: opioids’ effects on heterozygous patients. Int J Immunopathol Pharmacol 24:993–1004 22230405 10.1177/039463201102400417

[31] VanderVaart S, Berger H, Sistonen J, Madadi P, Matok I, Gijsen VM et al (2011) CYP2D6 polymorphisms and codeine analgesia in postpartum pain management: a pilot study. Ther Drug Monit 33:425–432 21743374 10.1097/FTD.0b013e3182272b10

[32] Camorcia M, Capogna G, Stirparo S, Berritta C, Blouin JL, Landau R (2012) Effect of μ-opioid receptor A118G polymorphism on the ED50 of epidural sufentanil for labor analgesia. Int J Obstet Anesth 21:40–44 22153130 10.1016/j.ijoa.2011.10.001

[33] Landau R, Liu SK, Blouin JL, Carvalho B (2013) The effect of OPRM1 and COMT genotypes on the analgesic response to intravenous fentanyl labor analgesia. Anesth Analg 116:386–391 23302985 10.1213/ANE.0b013e318273f2c7

[34] Ginosar Y, Birnbach DJ, Shirov TT, Arheart K, Caraco Y, Davidson EM (2013) Duration of analgesia and pruritus following intrathecal fentanyl for labour analgesia: no significant effect of A118G μ-opioid receptor polymorphism, but a marked effect of ethnically distinct hospital populations. Br J Anaesth 111:433–444 23592691 10.1093/bja/aet075

[35] Quinta R (2014) CYP2D6 genetic variation and predicted metabolic profile in post-cesarean section pain: pharmacogenetic interpretation. PhD Thesis. Universidade de Coimbra (Portugal)

[36] Stauble ME, Moore AW, Langman LJ, Boswell MV, Baumgartner R, McGee S et al (2014) Hydrocodone in postoperative personalized pain management: pro-drug or drug? Clin Chim Acta 429:26–29 24269714 10.1016/j.cca.2013.11.015

[37] Xu GH, Gao M, Sheng QY, Liu XS, Gu EW (2015) Opioid receptor A118G polymorphism does not affect the consumption of sufentanil and ropivacaine by patient-controlled epidural analgesia after cesarean section. Ther Drug Monit 37:53–57 24977380 10.1097/FTD.0000000000000112

[38] Pettini E, Micaglio M, Bitossi U, De Gaudio AR, Degl’Innocenti DR, Tofani L et al (2018) Influence of OPRM1 polymorphism on postoperative pain after intrathecal morphine administration in italian patients undergoing elective cesarean section. Clin J Pain 34:178–181 28591085 10.1097/AJP.0000000000000520

[39] Xie W, Zhuang W, Chen L, Xie W, Jiang C, Liu N (2018) 4218T/C polymorphism associations with post-cesarean patient-controlled epidural fentanyl consumption and pain perception. Acta Anaesthesiol Scand 62:376–383 29148033 10.1111/aas.13040

[40] Zgheib NK, Aouad MT, Taha SK, Nassar AH, Masri RF, Khoury MY et al (2018) μ-opioid receptor genetic polymorphisms and duration of epidural fentanyl analgesia during early labor. Minerva Anestesiol 84:946–954 29756748 10.23736/S0375-9393.18.12697-6

[41] Kung CC, Chen SS, Yang HJ, Lai CJ, Chen LK (2018) Pharmacogenetic study of pruritus induced by epidural morphine for post cesarean section analgesia. Taiwan J Obstet Gynecol 57:89–94 29458911 10.1016/j.tjog.2017.12.015

[42] Wang L, Wei C, Xiao F, Chang X, Zhang Y (2019) Influences of COMT rs4680 and OPRM1 rs1799971 polymorphisms on chronic postsurgical pain, acute pain, and analgesic consumption after elective cesarean delivery. Clin J Pain 35:31–36 30234521 10.1097/AJP.0000000000000654

[43] Xiaohong Y, Xue-ming H (2020) COMTval158met gene polymorphism correlation with prenatal anxiety and labor analgesia. Int J Hum Genet 20:104–109

[44] Shu X, Yan Y, Yu J, Chi L (2024) Cytochrome P4503A4 gene polymorphisms guide safe sufentanil analgesic doses in pregnant Chinese mothers: a multicenter, randomized, prospective study. Pharmacogenet Genomics 34:8–15 37962984 10.1097/FPC.0000000000000513

[45] Li W, Xiao T, Wu X, Wu X, Xiang R, Liu H et al (2024) Association between ABCB1 gene polymorphisms and labor analgesia in primiparas. Clin Exp Obstet Gynecol 51:200

[46] Walter C, Doehring A, Oertel BG, Lötsch J (2013) µ-opioid receptor gene variant OPRM1 118 A>G: a summary of its molecular and clinical consequences for pain. Pharmacogenomics 14:1915–1925 10.2217/pgs.13.187 24236490

[47] Mestek A, Hurley JH, Bye LS, Campbell AD, Chen Y, Tian M et al (1995) The human mu opioid receptor: modulation of functional desensitization by calcium/calmodulin-dependent protein kinase and protein kinase C. J Neurosci 15:2396–2406 7891175 10.1523/JNEUROSCI.15-03-02396.1995 PMC6578163

[48] Bond C, LaForge KS, Tian M, Melia D, Zhang S, Borg L et al (1998) Single-nucleotide polymorphism in the human mu opioid receptor gene alters beta-endorphin binding and activity: possible implications for opiate addiction. Proc Natl Acad Sci U S A 95:9608–9613 9689128 10.1073/pnas.95.16.9608 PMC21386

[49] Oertel BG, Kettner M, Scholich K, Renné C, Roskam B, Geisslinger G et al (2009) A common human micro-opioid receptor genetic variant diminishes the receptor signaling efficacy in brain regions processing the sensory information of pain. J Biol Chem 284:6530–6535 19116204 10.1074/jbc.M807030200

[50] Zhang Y, Wang D, Johnson AD, Papp AC, Sadée W (2005) Allelic expression imbalance of human mu opioid receptor (OPRM1) caused by variant A118G. J Biol Chem 280:32618–32624 16046395 10.1074/jbc.M504942200

[51] Oertel BG, Doehring A, Roskam B, Kettner M, Hackmann N, Ferreirós N et al (2012) Genetic-epigenetic interaction modulates μ-opioid receptor regulation. Hum Mol Genet 21:4751–4760 22875838 10.1093/hmg/dds314

[52] Crews KR, Monte AA, Huddart R, Caudle KE, Kharasch ED, Gaedigk A et al (2021) Clinical pharmacogenetics implementation consortium guideline for CYP2D6, OPRM1, and COMT genotypes and select opioid therapy. Clin Pharmacol Ther 110:888–896 33387367 10.1002/cpt.2149 PMC8249478

[53] Song Z, Du B, Wang K, Shi X (2013) Effects of OPRM1 A118G polymorphism on epidural analgesia with fentanyl during labor: a meta-analysis. Genet Test Mol Biomarkers 17:743–749 23909491 10.1089/gtmb.2013.0282

[54] Li ZX, Ye F, Li WY, Bao YP, Cheng YC, Song ZW et al (2023) The effect of genetic variation on the sensitivity to opioid analgesics in patients with postoperative pain: an updated meta-analysis. Pain Physician 26:E467–E485 37774182

[55] Frangakis SG, MacEachern M, Akbar TA, Bolton C, Lin V, Smith AV et al (2023) Association of genetic variants with postsurgical pain: a systematic review and meta-analyses. Anesthesiology 139:827–839 37774411 10.1097/ALN.0000000000004677 PMC10859728

[56] Landau R, Ortner CM, Vuilleumier PH (2013) The impact of genetics and other factors on intra- and post-partum pain. Curr Anesthesiol Rep 3:264–274

[57] Kong Y, Yan T, Gong S, Deng H, Zhang G, Wang J (2018) Opioid receptor mu 1 (OPRM1) A118G polymorphism (rs1799971) and postoperative nausea and vomiting. Am J Transl Res 10:2764–2780 30323865 PMC6176240

[58] Zhang X, Liang Y, Zhang N, Yan Y, Liu S, Fengxi H et al (2019) The relevance of the OPRM1 118A>G genetic variant for opioid requirement in pain treatment: a meta-analysis. Pain Physician 22:331–340 31337162

