# Supplementary material for: Pharmacogenetics of opioid medications for relief of labor pain and post-cesarean pain: a systematic review and meta-analysis
Source: Eur J Clin Pharmacol. 2025 Jan 7;81(3):403–17. doi: 10.1007/s00228-024-03798-z (PMC11825576; doi:10.1007/s00228-024-03798-z)
Supplement: Supplementary file 1 — Supplementary file1 (PDF 532 KB) [file 228_2024_3798_MOESM1_ESM.pdf]

Supplementary Table 1. MOOSE Checklist for Meta-analyses of Observational Studies.

Item No	Recommendation	Reported on Page No
Reporting of background should include		
1	Problem definition	3
2	Hypothesis statement	3
3	Description of study outcome(s)	3
4	Type of exposure or intervention used	3
5	Type of study designs used	3
6	Study population	3
Reporting of search strategy should include		
7	Qualifications of searchers (eg, librarians and investigators)	4
8	Search strategy, including time period included in the synthesis and key words	4
9	Effort to include all available studies, including contact with authors	4
10	Databases and registries searched	4
11	Search software used, name and version, including special features used (eg, explosion)	-
12	Use of hand searching (eg, reference lists of obtained articles)	4
13	List of citations located and those excluded, including justification	5
14	Method of addressing articles published in languages other than English	4
15	Method of handling abstracts and unpublished studies	4
16	Description of any contact with authors	4
Reporting of methods should include		
17	Description of relevance or appropriateness of studies assembled for assessing the hypothesis to be tested	4
18	Rationale for the selection and coding of data (eg, sound clinical principles or convenience)	5
19	Documentation of how data were classified and coded (eg, multiple raters, blinding and interrater reliability)	5
20	Assessment of confounding (eg, comparability of cases and controls in studies where appropriate)	6
21	Assessment of study quality, including blinding of quality assessors, stratification or regression on possible predictors of study results	6
22	Assessment of heterogeneity	6
23	Description of statistical methods (eg, complete description of fixed or random effects models, justification of whether the chosen models account for predictors of study results, dose-response models, or cumulative meta-analysis) in sufficient detail to be replicated	6-7
24	Provision of appropriate tables and graphics	6
Reporting of results should include		
25	Graphic summarizing individual study estimates and overall estimate	Fig. 2-4,<br>Suppl. Fig. 4-11
26	Table giving descriptive information for each study included	Table 1
27	Results of sensitivity testing (eg, subgroup analysis)	Fig 2-4,<br>Suppl. Fig. 2, 4, 5-11

28	Indication of statistical uncertainty of findings	Fig 2-4<br>Suppl Fig 1-11

Item No	Recommendation	Reported on Page No
Reporting of discussion should include		
29	Quantitative assessment of bias (eg, publication bias)	12
30	Justification for exclusion (eg, exclusion of non-English language citations)	12
31	Assessment of quality of included studies	10, 12
Reporting of conclusions should include		
32	Consideration of alternative explanations for observed results	12-13
33	Generalization of the conclusions (ie, appropriate for the data presented and within the domain of the literature review)	13
34	Guidelines for future research	13
35	Disclosure of funding source	14

From: Stroup DF, Berlin JA, Morton SC, et al, for the Meta-analysis Of Observational Studies in Epidemiology (MOOSE) Group. Meta-analysis of Observational Studies in Epidemiology. A Proposal for Reporting. *JAMA*. 2000;283(15):2008-2012. doi: 10.1001/jama.283.15.2008.

Supplementary Fig. 1

Trim-and-fill funnel plot of the standardized mean difference of pain score after opioid treatment for relief of labor pain and post-cesarean pain, for the dominant model of *OPRM1* rs1799971 (GG or AG vs AA) (adjusted SMD: 0.18; 95% CI 0.08–0.28, P = 0.001).

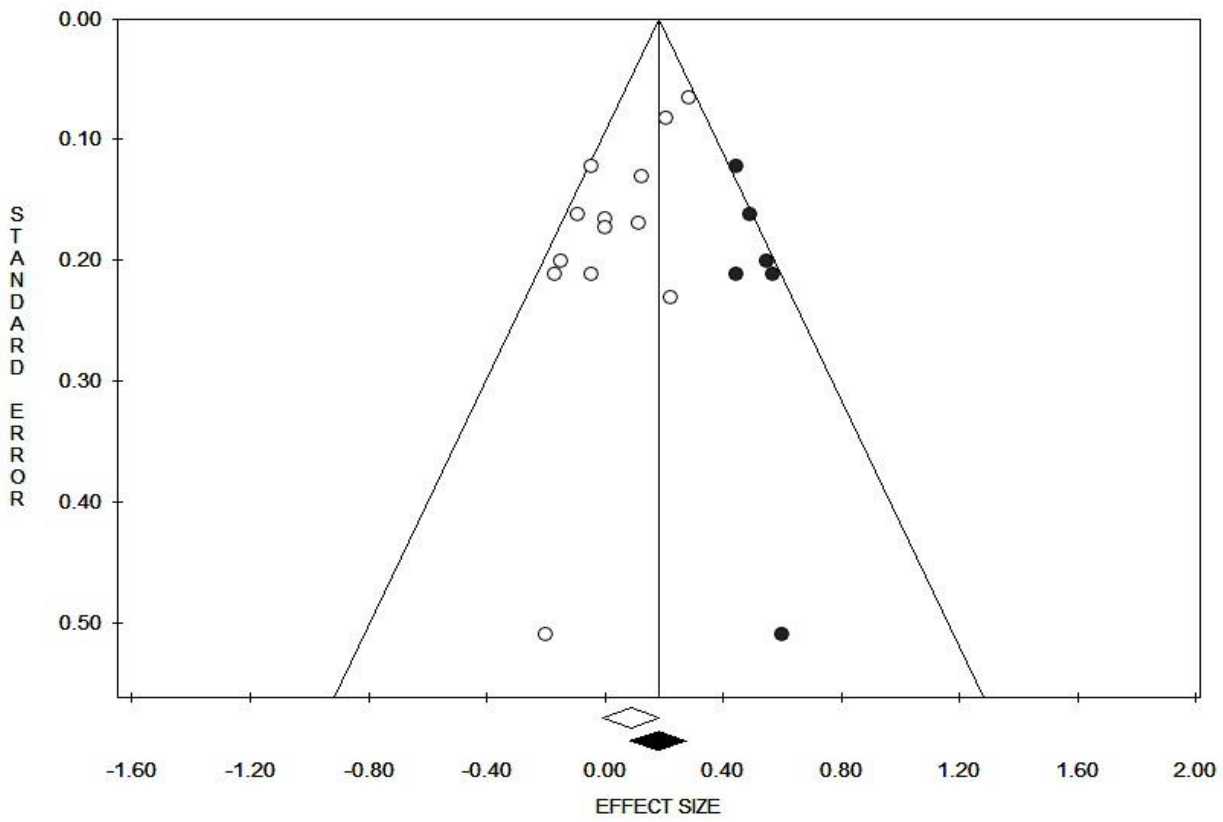

Supplementary Fig. 2

Leave-one-out meta-analyses for the standardized mean differences of total opioid consumption after opioid treatment for relief of labor pain and post-cesarean pain, for the dominant (GG or AG vs AA) (**A**) or the recessive model (GG vs AG or AA) (**B**) of *OPRM1* rs1799971.

A

	ES	95% CI	Sig.	N1	N2
Tan E 2009	0.30	0.17 , 0.43	0.000	605	389
Sia AT 2008	0.30	0.20 , 0.40	0.000	919	660
Chen Y 2023	0.30	0.21 , 0.39	0.000	1055	796
Wang L 2019	0.34	0.24 , 0.43	0.000	1181	936
Zhang J 2018	0.46	0.26 , 0.67	0.000	1319	1038
Wong CA 2010	0.42	0.23 , 0.61	0.000	1365	1182
Xu GH 2015	0.36	0.16 , 0.55	0.000	1463	1245
Boswell MV 2013	0.30	0.11 , 0.50	0.002	1490	1375
Wong CA. 2010	0.28	0.09 , 0.46	0.003	1515	1453
Baber M 2015	0.26	0.09 , 0.44	0.003	1550	1516

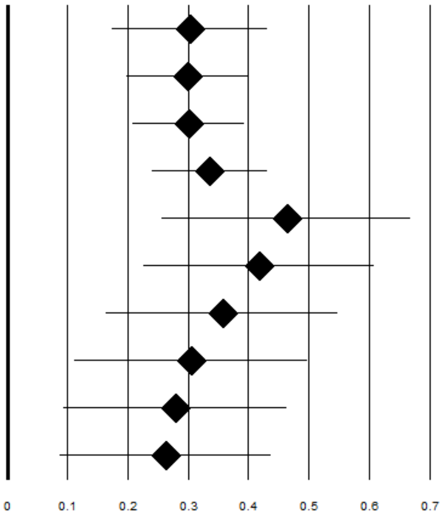

B

	ES	95% CI	Sig.	N1	N2
Baber M 2015	0.67	0.20 , 1.14	0.005	329	1917
Sia AT 2008	0.65	0.06 , 1.24	0.030	256	1503
Tan E 2009	0.68	0.14 , 1.23	0.014	166	1184
Wang L 2019	0.49	0.04 , 0.94	0.031	305	1773
Xu GH 2015	0.65	0.16 , 1.14	0.010	319	1864
Zhang J 2018	0.41	0.09 , 0.72	0.011	305	1799

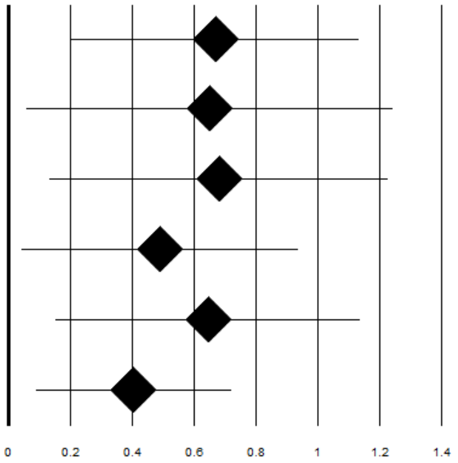

Supplementary Fig. 3

Funnel plot of the standardized mean difference of total opioid consumption after opioid treatment for relief of labor pain and post-cesarean pain, for the dominant model of *OPRM1* rs1799971 (GG or AG vs AA). Egger's test P-value = 0.431.

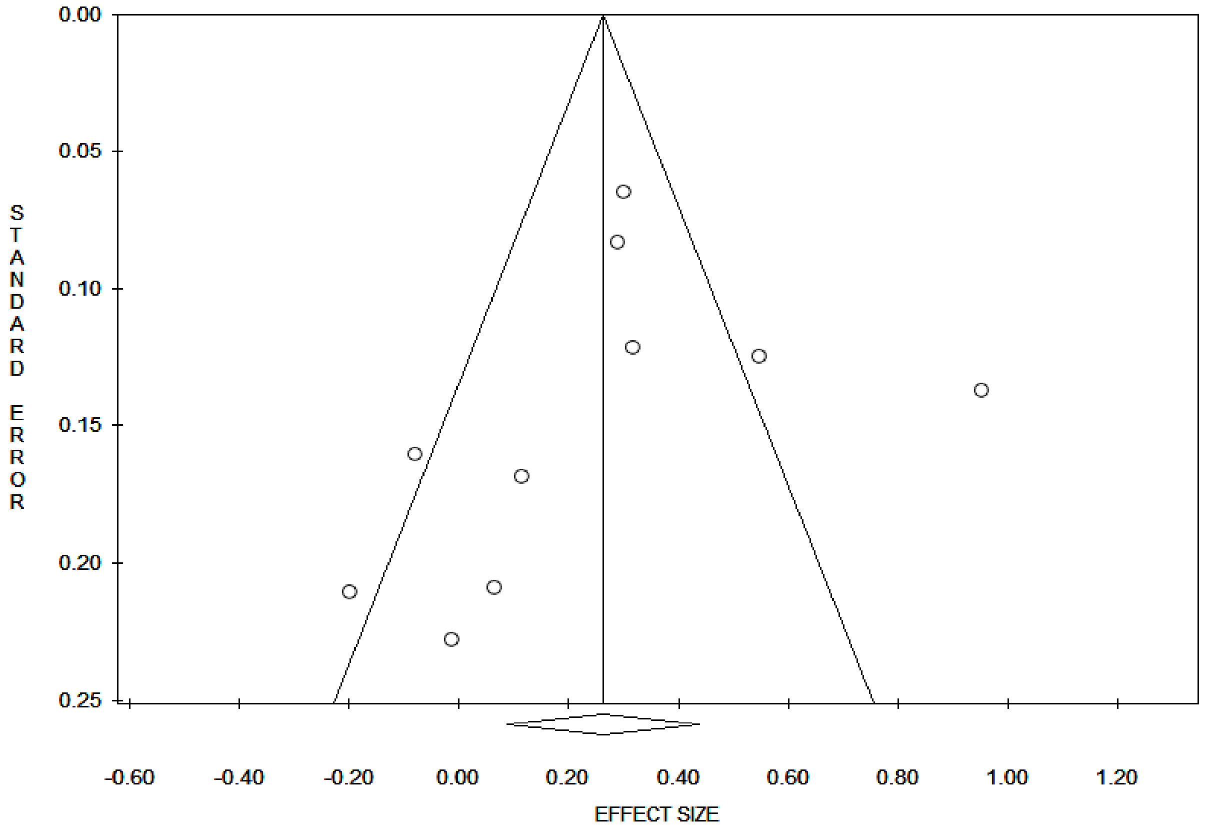

Supplementary Fig. 4

Forest plot for the dominant (GG or AG vs AA) (**A**) or the recessive (GG vs AG or AA) (**B**) model of *OPRM1* rs1799971 for the risk of pruritus following opioid treatment. Note that the diamond symbol in B is shown twice to emphasize the lack of studies for the subgroup of patients with labor pain.

A

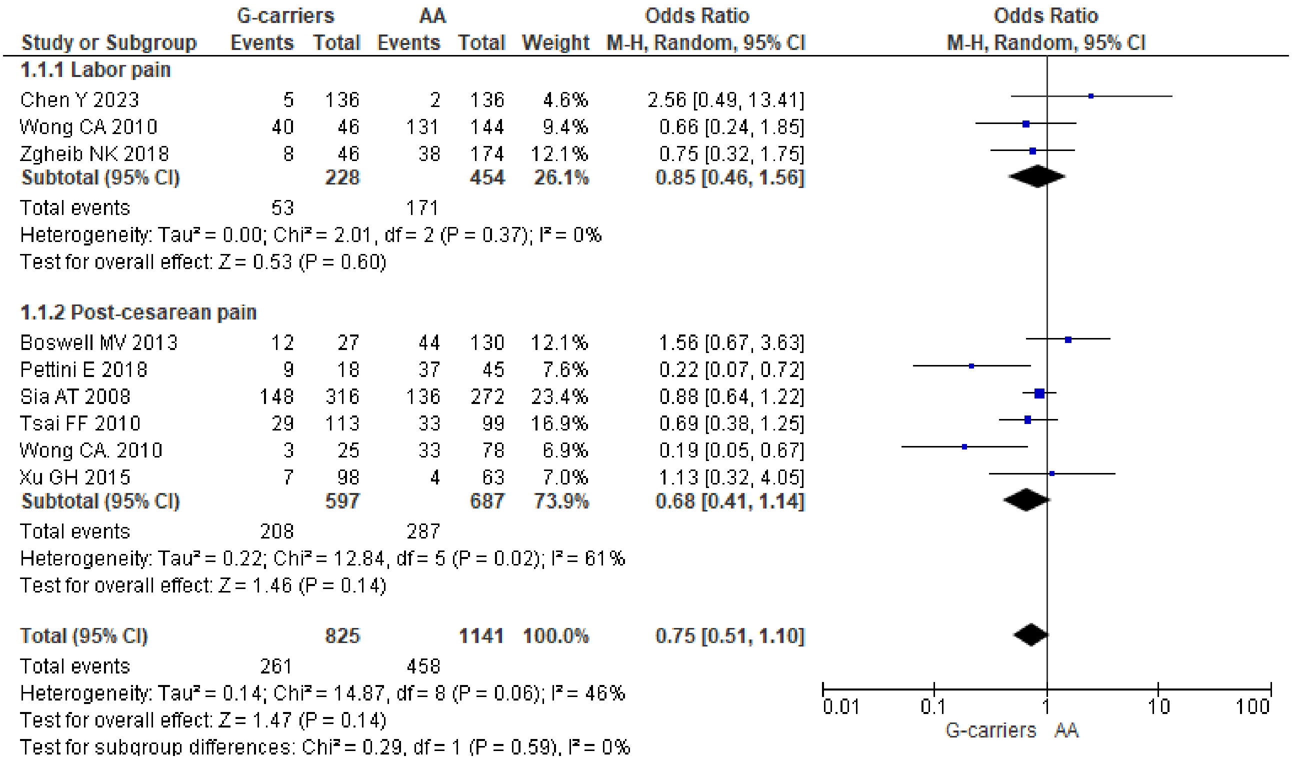

B

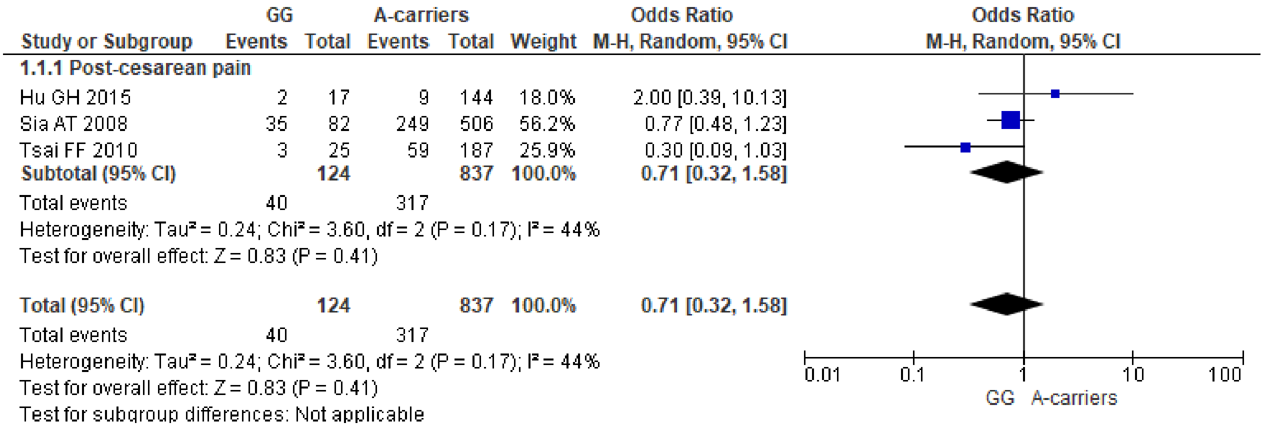

Supplementary Fig. 5

Forest plot for the dominant (GG or AG vs AA) (**A**) or the recessive (GG vs AG or AA) (**B**) model of *OPRM1* rs1799971 for the risk of nausea following opioid treatment for relief of labor pain and post-cesarean pain. Note that the diamond symbol in B is shown twice to emphasize the lack of studies for the subgroup of patients with labor pain.

A

Study or Subgroup	G-carriers Events	G-carriers Total	AA Events	AA Total	Weight	Odds Ratio M-H, Random, 95% CI
**1.1.1 Labor pain**						
Wong CA 2010	1	46	11	144	4.6%	0.27 [0.03, 2.14]
Zgheib NK 2018	3	46	4	174	8.0%	2.97 [0.64, 13.75]
**Subtotal (95% CI)**		**92**		**318**	**12.6%**	**0.98 [0.08, 11.44]**
Total events	4		15			
Heterogeneity: Tau² = 2.29; Chi² = 3.64, df = 1 (P = 0.06); I² = 73%						
Test for overall effect: Z = 0.01 (P = 0.99)						
**1.1.2 Post-cesarean pain**						
Boswell MV 2013	6	27	27	130	15.9%	1.09 [0.40, 2.97]
Sia AT 2008	14	316	26	272	26.7%	0.44 [0.22, 0.86]
Wong CA. 2010	1	25	3	78	3.8%	1.04 [0.10, 10.49]
Xu GH 2015	8	98	5	63	12.6%	1.03 [0.32, 3.31]
Zhang J 2018	28	138	21	102	28.4%	0.98 [0.52, 1.85]
**Subtotal (95% CI)**		**604**		**645**	**87.4%**	**0.77 [0.52, 1.14]**
Total events	57		82			
Heterogeneity: Tau² = 0.00; Chi² = 4.04, df = 4 (P = 0.40); I² = 1%						
Test for overall effect: Z = 1.33 (P = 0.18)						
**Total (95% CI)**		**696**		**963**	**100.0%**	**0.83 [0.52, 1.33]**
Total events	61		97			
Heterogeneity: Tau² = 0.09; Chi² = 7.98, df = 6 (P = 0.24); I² = 25%						
Test for overall effect: Z = 0.76 (P = 0.45)						
Test for subgroup differences: Chi² = 0.04, df = 1 (P = 0.85), I² = 0%						

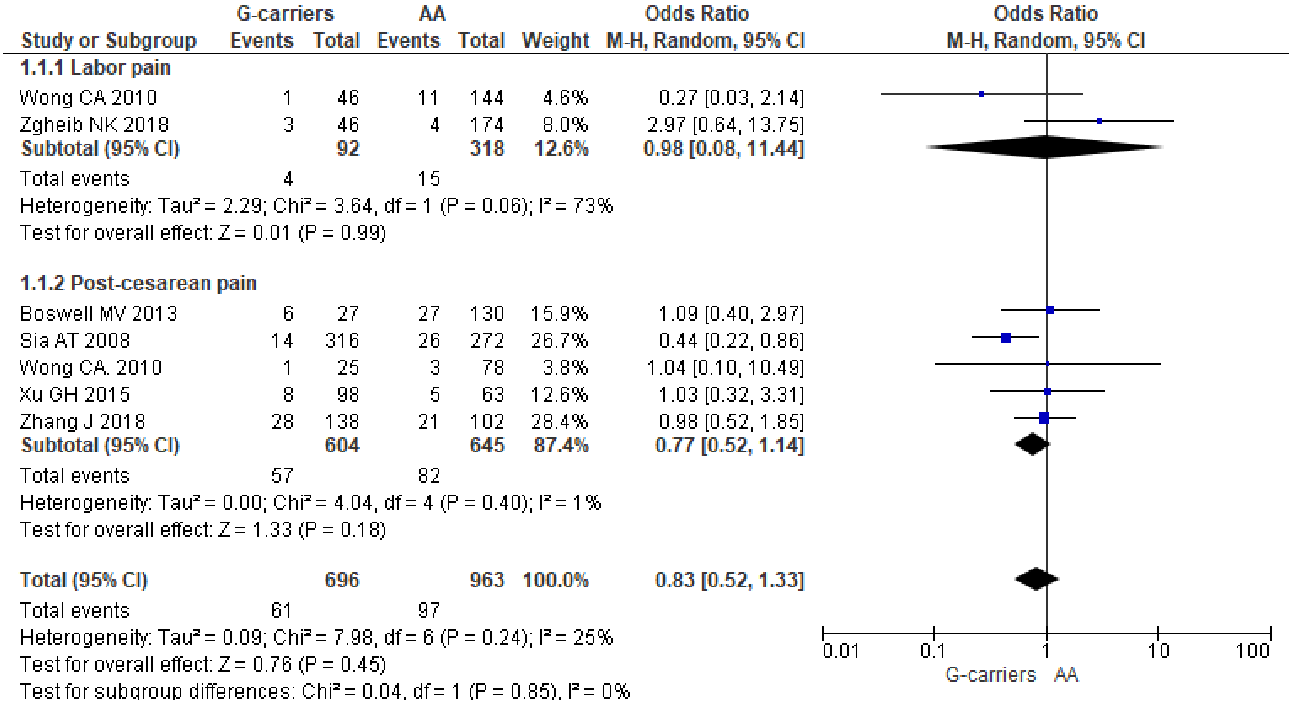

B

Study or Subgroup	GG Events	GG Total	A-carriers Events	A-carriers Total	Weight	Odds Ratio M-H, Random, 95% CI
**1.1.1 Post-cesarean pain**						
Sia AT 2008	1	82	39	506	29.8%	0.15 [0.02, 1.09]
Xu GH 2015	2	17	11	14	30.3%	0.04 [0.01, 0.26]
Zhang J 2018	5	31	44	209	39.9%	0.72 [0.26, 1.99]
**Subtotal (95% CI)**		**130**		**729**	**100.0%**	**0.18 [0.03, 1.18]**
Total events	8		94			
Heterogeneity: Tau² = 2.02; Chi² = 7.91, df = 2 (P = 0.02); I² = 75%						
Test for overall effect: Z = 1.78 (P = 0.07)						
**Total (95% CI)**		**130**		**729**	**100.0%**	**0.18 [0.03, 1.18]**
Total events	8		94			
Heterogeneity: Tau² = 2.02; Chi² = 7.91, df = 2 (P = 0.02); I² = 75%						
Test for overall effect: Z = 1.78 (P = 0.07)						
Test for subgroup differences: Not applicable						

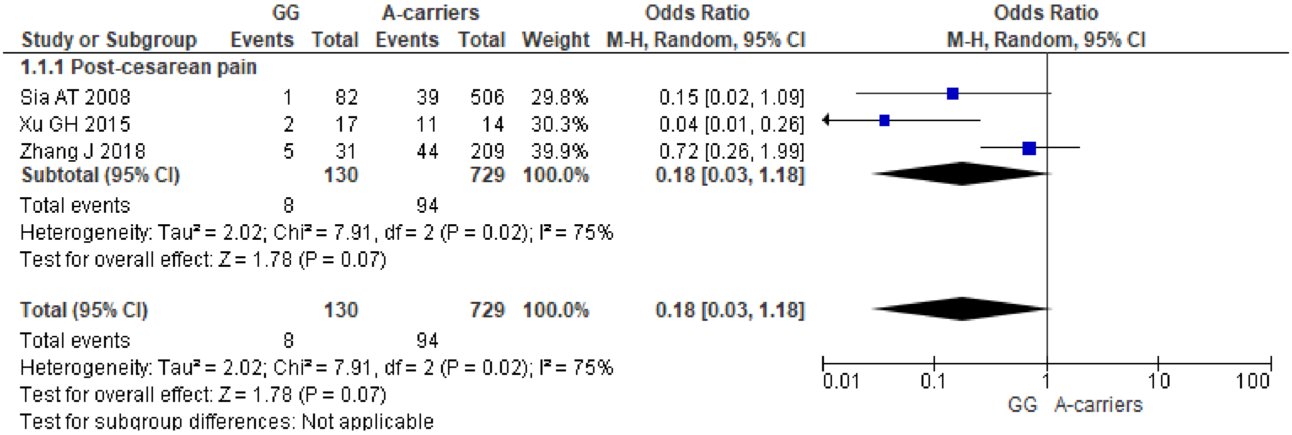

Supplementary Fig. 6

Forest plot for the dominant model of *OPRM1* rs1799971 (GG or AG vs AA) for the risk of vomiting following opioid treatment for relief of labor pain and post-cesarean pain.

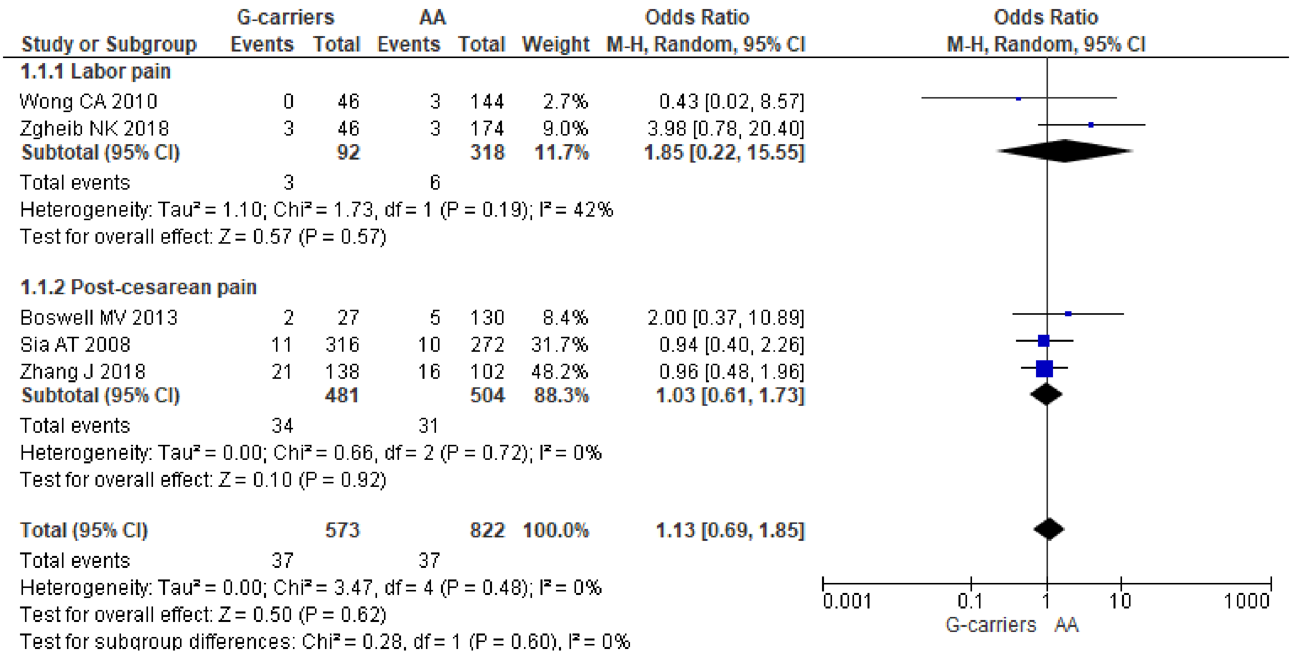

Supplementary Results

Quantitative data synthesis for *COMT* rs4680

Four studies were included in the meta-analysis of association between the dominant model of *COMT* rs4680 and total opioid consumption [17, 23, 42, 43], while three studies only were available for the recessive model [23, 42, 43]. In the overall pooled analyses, no significant impact of *COMT* rs4680 was found on total opioid consumption, under either the dominant (Supplementary Fig. 7A) or the recessive genetic model (Supplementary Fig. 7B). Despite a significant difference in total opioid consumption was detected under the dominant model of *COMT* rs4680 within the subgroup of patients with labor pain (GA+AA vs GG, SMD: 0.51; 95% CI: 0.05-0.98; P= 0.03, Supplementary Fig. 7A), this result was limited by significant between-study heterogeneity (I^2: 76%, P=0.04).

Quantitative data synthesis for other genetic variants

Meta-analyses with at least three studies were available for the association of *ABCB1* rs1128503 and *CYP3A4* rs2242480 with both pain score after opioid treatment and total opioid consumption, and three studies were available for the association of the genetically predicted *CYP2D6* phenotype with pain score. In all cases, no significant results were found in the pooled analyses, as shown in Supplementary Fig. 8-12.

Supplementary Fig. 7

Forest plots of the standardized mean differences of total opioid consumption after opioid treatment for relief of labor pain and post-cesarean pain, for the dominant (GA+AA vs GG) (**A**) or the recessive model (AA vs GA+GG) (**B**) of *COMT* rs4680.

A

Study or Subgroup	A-carriers Mean	A-carriers SD	A-carriers Total	GG Mean	GG SD	GG Total	Weight	Std. Mean Difference IV, Random, 95% CI
**1.1.1 Labor pain**								
Chen Y 2023	16.8	8.63	173	14.26	7.84	183	27.7%	0.31 [0.10, 0.52]
Xiaohong Y 2020	53.95	7.6	52	47.85	7.79	45	22.7%	0.79 [0.37, 1.20]
**Subtotal (95% CI)**			**225**			**228**	**50.4%**	**0.51 [0.05, 0.98]**
Heterogeneity: Tau² = 0.09; Chi² = 4.09, df = 1 (P = 0.04); I² = 76%								
Test for overall effect: Z = 2.16 (P = 0.03)								
**1.1.2 Post-cesarean pain**								
Baber M 2015	2.36	2.99	65	2.25	2.76	33	22.6%	0.04 [-0.38, 0.46]
Wang L 2019	98.35	11.57	136	101.4	13.8	130	27.0%	-0.24 [-0.48, 0.00]
**Subtotal (95% CI)**			**201**			**163**	**49.6%**	**-0.16 [-0.40, 0.09]**
Heterogeneity: Tau² = 0.01; Chi² = 1.26, df = 1 (P = 0.26); I² = 21%								
Test for overall effect: Z = 1.23 (P = 0.22)								
**Total (95% CI)**			**426**			**391**	**100.0%**	**0.21 [-0.19, 0.61]**
Heterogeneity: Tau² = 0.14; Chi² = 21.53, df = 3 (P < 0.0001); I² = 86%								
Test for overall effect: Z = 1.01 (P = 0.31)								
Test for subgroup differences: Chi² = 6.18, df = 1 (P = 0.01), I² = 83.8%								

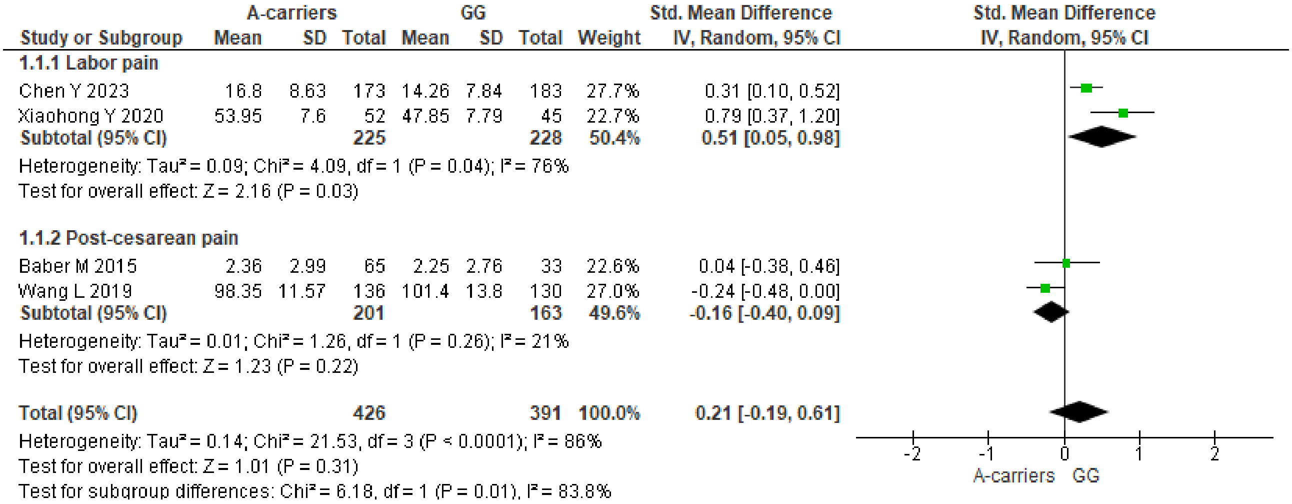

B

Study or Subgroup	AA Mean	AA SD	AA Total	G-carriers Mean	G-carriers SD	G-carriers Total	Weight	Std. Mean Difference IV, Random, 95% CI
**1.1.1 Labor pain**								
Xiaohong Y 2020	57.32	7.48	14	50.07	7.92	83	31.9%	0.91 [0.33, 1.50]
**Subtotal (95% CI)**			**14**			**83**	**31.9%**	**0.91 [0.33, 1.50]**
Heterogeneity: Not applicable								
Test for overall effect: Z = 3.08 (P = 0.002)								
**1.1.2 Post-cesarean pain**								
Baber M 2015	2.54	2.7	14	2.29	2.95	84	32.2%	0.09 [-0.48, 0.65]
Wang L 2019	95.7	11.6	30	100.37	12.84	236	35.9%	-0.37 [-0.75, 0.01]
**Subtotal (95% CI)**			**44**			**320**	**68.1%**	**-0.19 [-0.62, 0.24]**
Heterogeneity: Tau² = 0.04; Chi² = 1.68, df = 1 (P = 0.19); I² = 41%								
Test for overall effect: Z = 0.87 (P = 0.38)								
**Total (95% CI)**			**58**			**403**	**100.0%**	**0.19 [-0.56, 0.93]**
Heterogeneity: Tau² = 0.37; Chi² = 13.09, df = 2 (P = 0.001); I² = 85%								
Test for overall effect: Z = 0.49 (P = 0.62)								
Test for subgroup differences: Chi² = 8.97, df = 1 (P = 0.003), I² = 88.9%								

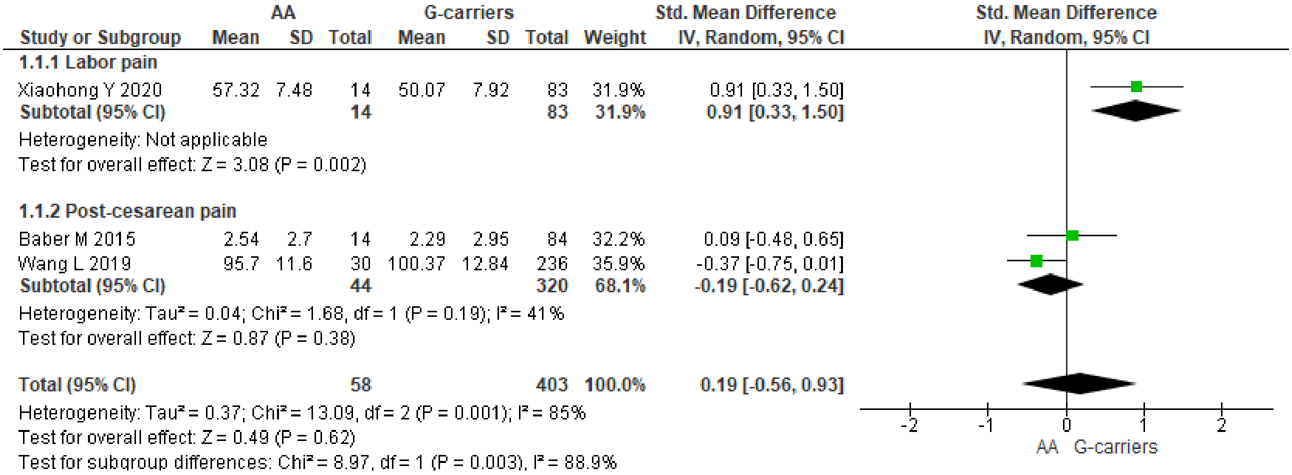

Supplementary Fig. 8

Forest plots of the standardized mean differences of pain score after opioid treatment for relief of labor pain and post-cesarean pain, for the dominant (CT+TT vs CC) (**A**) or the recessive model (TT vs CT+CC) (**B**) of *ABCB1* rs1128503.

A

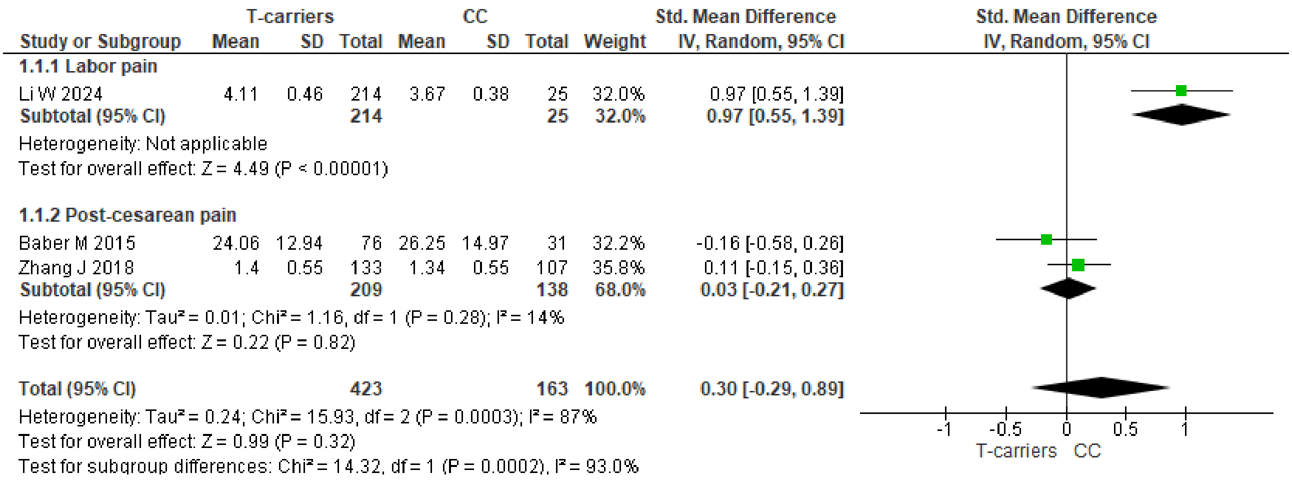

B

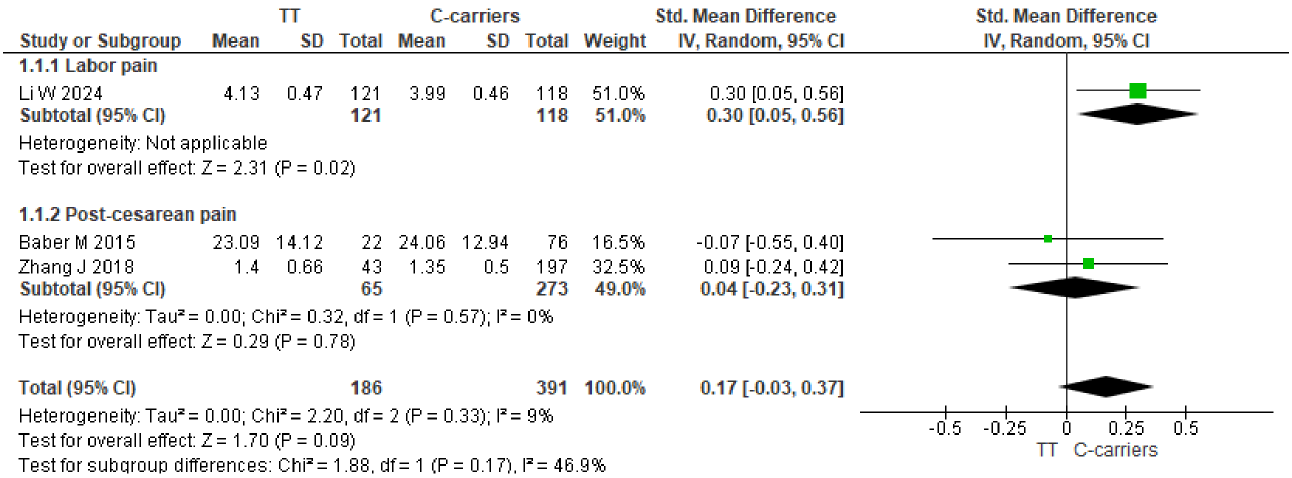

Supplementary Fig. 9

Forest plots of the standardized mean differences of total opioid consumption after opioid treatment for relief of post-cesarean pain, for the dominant (CT+TT vs CC) (**A**) or the recessive model (TT vs CT+CC) (**B**) of *ABCB1* rs1128503. Note that the diamond symbol in A and B is shown twice to emphasize the lack of studies for the subgroup of patients with labor pain.

A

	T-carriers			CC				Std. Mean Difference
**Study or Subgroup**	**Mean**	**SD**	**Total**	**Mean**	**SD**	**Total**	**Weight**	**IV, Random, 95% CI**
**1.1.1 Post-cesarean pain**								
Baber M 2015	2.43	2.84	67	2.26	2.56	31	27.5%	0.06 [-0.36, 0.49]
Sia AT 2010	7.11	8.12	551	8.4	8.7	61	36.1%	-0.16 [-0.42, 0.11]
Zhang J 2018	10.01	3.84	133	8.8	2.08	107	36.5%	0.38 [0.12, 0.64]
**Subtotal (95% CI)**			**751**			**199**	**100.0%**	**0.10 [-0.26, 0.46]**
Heterogeneity: Tau² = 0.07; Chi² = 8.21, df = 2 (P = 0.02); I² = 76%								
Test for overall effect: Z = 0.54 (P = 0.59)								
**Total (95% CI)**			**751**			**199**	**100.0%**	**0.10 [-0.26, 0.46]**
Heterogeneity: Tau² = 0.07; Chi² = 8.21, df = 2 (P = 0.02); I² = 76%								
Test for overall effect: Z = 0.54 (P = 0.59)								
Test for subgroup differences: Not applicable								

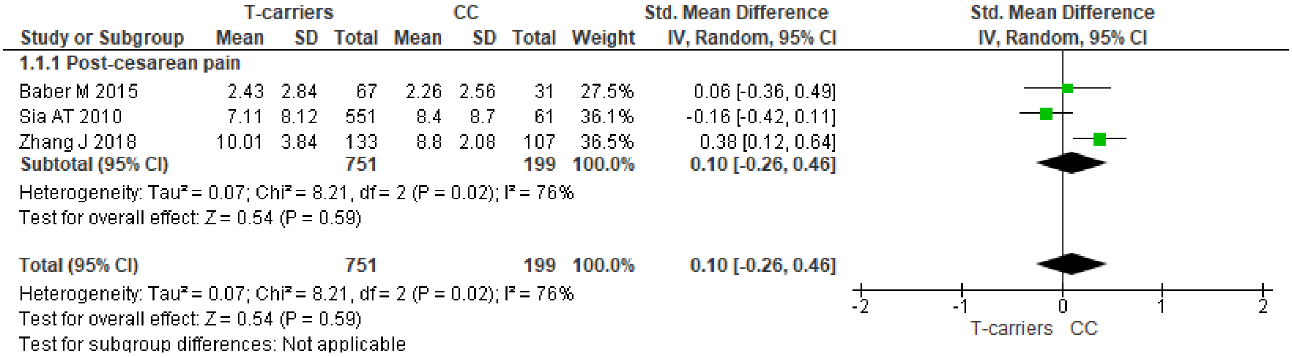

B

	TT			C-carriers				Std. Mean Difference
**Study or Subgroup**	**Mean**	**SD**	**Total**	**Mean**	**SD**	**Total**	**Weight**	**IV, Random, 95% CI**
**1.1.1 Post-cesarean pain**								
Baber M 2015	2.76	3.17	22	2.27	2.62	76	30.2%	0.18 [-0.30, 0.65]
Sia AT 2010	7.3	8.5	288	7.18	7.91	324	36.5%	0.01 [-0.14, 0.17]
Zhang J 2018	11.77	4.02	43	8.97	2.85	197	33.3%	0.90 [0.56, 1.24]
**Subtotal (95% CI)**			**353**			**597**	**100.0%**	**0.36 [-0.23, 0.95]**
Heterogeneity: Tau² = 0.24; Chi² = 21.58, df = 2 (P < 0.0001); I² = 91%								
Test for overall effect: Z = 1.20 (P = 0.23)								
**Total (95% CI)**			**353**			**597**	**100.0%**	**0.36 [-0.23, 0.95]**
Heterogeneity: Tau² = 0.24; Chi² = 21.58, df = 2 (P < 0.0001); I² = 91%								
Test for overall effect: Z = 1.20 (P = 0.23)								
Test for subgroup differences: Not applicable								

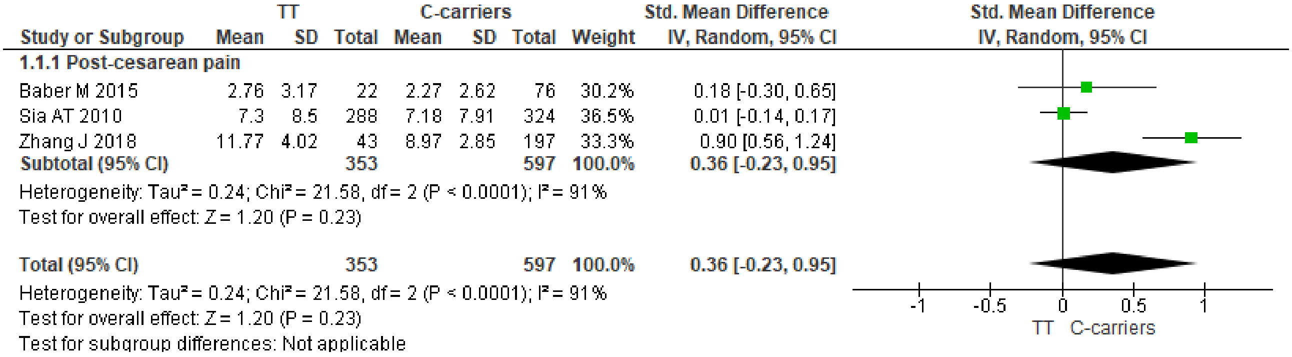

Supplementary Fig. 10

Forest plot of the standardized mean difference of pain score after opioid treatment for relief of labor pain and post-cesarean pain, for the dominant model of *CYP3A4* rs2242480 (GA+AA vs GG).

	A-carriers			GG				Std. Mean Difference
Study or Subgroup	Mean	SD	Total	Mean	SD	Total	Weight	IV, Random, 95% CI
**1.1.1 Labor pain**								
Shu X 2024	2.02	1.24	184	1.94	1.41	243	74.4%	0.06 [-0.13, 0.25]
**Subtotal (95% CI)**			**184**			**243**	**74.4%**	**0.06 [-0.13, 0.25]**
Heterogeneity: Not applicable								
Test for overall effect: Z = 0.61 (P = 0.54)								
**1.1.2 Post-cesarean pain**								
Lv J 2018	0.91	2.06	183	0.95	1.03	25	15.6%	-0.02 [-0.44, 0.40]
Zhang J 2018	1.43	0.56	225	1.4	0.63	15	10.0%	0.05 [-0.47, 0.58]
**Subtotal (95% CI)**			**408**			**40**	**25.6%**	**0.01 [-0.32, 0.33]**
Heterogeneity: Tau² = 0.00; Chi² = 0.05, df = 1 (P = 0.83); I² = 0%								
Test for overall effect: Z = 0.05 (P = 0.96)								
**Total (95% CI)**			**592**			**283**	**100.0%**	**0.05 [-0.12, 0.21]**
Heterogeneity: Tau² = 0.00; Chi² = 0.12, df = 2 (P = 0.94); I² = 0%								
Test for overall effect: Z = 0.55 (P = 0.58)								
Test for subgroup differences: Chi² = 0.07, df = 1 (P = 0.79), I² = 0%								

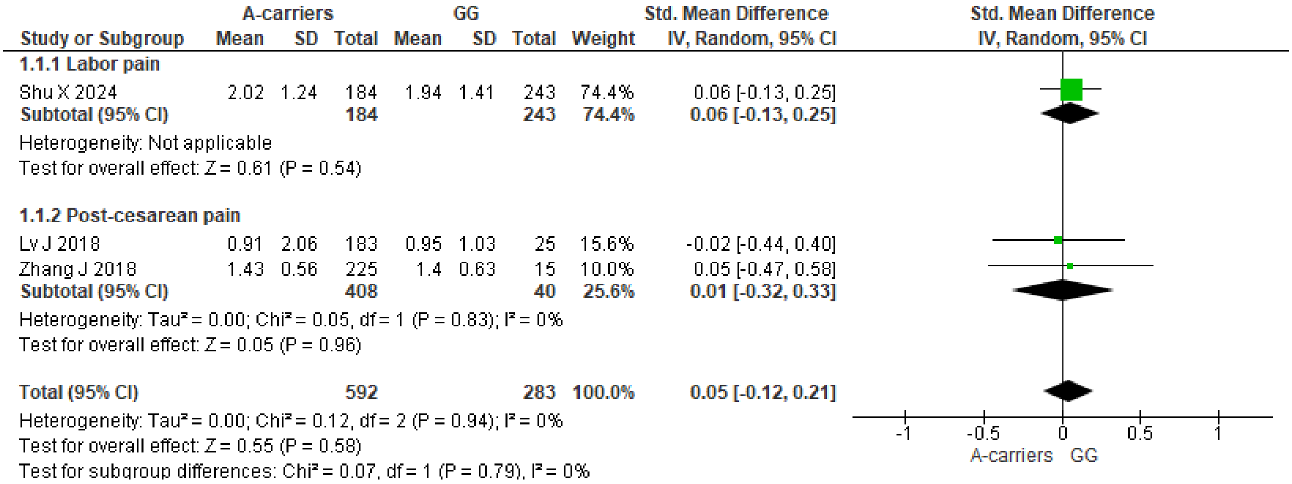

Supplementary Fig. 11

Forest plot of the standardized mean differences of total opioid consumption after opioid treatment for relief of labor pain and post-cesarean pain, for the dominant model of *CYP3A4* rs2242480 (GA+AA vs GG).

	A-carriers			GG				Std. Mean Difference
**Study or Subgroup**	**Mean**	**SD**	**Total**	**Mean**	**SD**	**Total**	**Weight**	**IV, Random, 95% CI**
**1.1.1 Labor pain**								
Shu X 2024	53.58	28.51	184	54.89	27.09	243	38.1%	-0.05 [-0.24, 0.14]
**Subtotal (95% CI)**			**184**			**243**	**38.1%**	**-0.05 [-0.24, 0.14]**
Heterogeneity: Not applicable								
Test for overall effect: Z = 0.48 (P = 0.63)								
**1.1.2 Post-cesarean pain**								
Lv J 2018	167.46	204.21	183	125.12	58.4	25	32.6%	0.22 [-0.20, 0.64]
Zhang J 2018	10.67	3.17	225	7.54	2.03	15	29.3%	1.00 [0.47, 1.53]
**Subtotal (95% CI)**			**408**			**40**	**61.9%**	**0.59 [-0.17, 1.36]**
Heterogeneity: Tau² = 0.25; Chi² = 5.16, df = 1 (P = 0.02); I² = 81%								
Test for overall effect: Z = 1.51 (P = 0.13)								
**Total (95% CI)**			**592**			**283**	**100.0%**	**0.35 [-0.21, 0.91]**
Heterogeneity: Tau² = 0.20; Chi² = 13.68, df = 2 (P = 0.001); I² = 85%								
Test for overall effect: Z = 1.22 (P = 0.22)								
Test for subgroup differences: Chi² = 2.52, df = 1 (P = 0.11), I² = 60.3%								

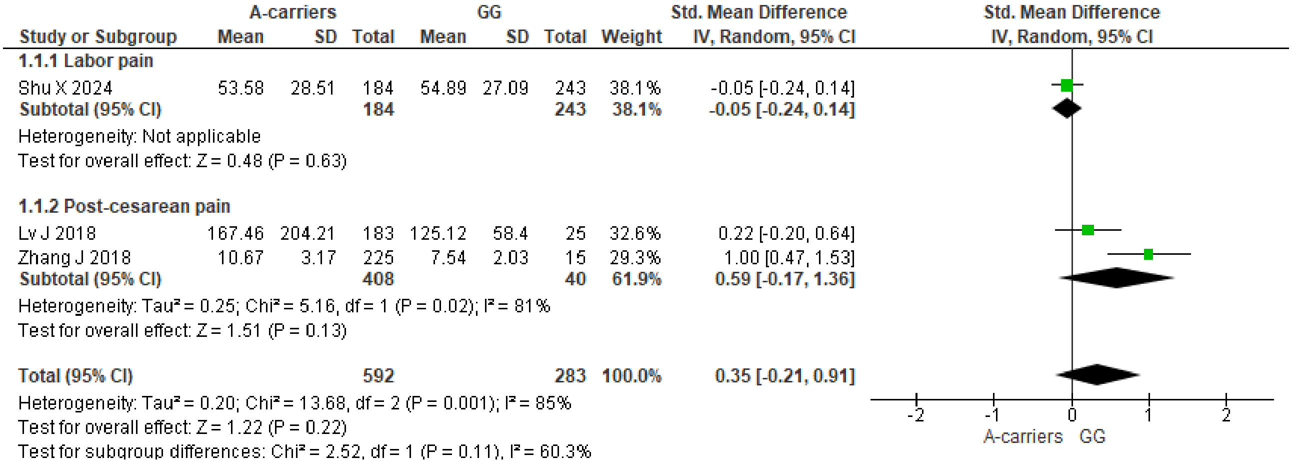

Supplementary Fig. 12

Forest plot of the mean difference of pain score after opioid treatment for relief of labor pain and post-cesarean pain, for *CYP2D6* (PM or IM vs UM or EM). Note that the diamond symbol is shown twice to emphasize the lack of studies for the subgroup of patients with labor pain.

	PM+IM			UM+EM				Std. Mean Difference
**Study or Subgroup**	**Mean**	**SD**	**Total**	**Mean**	**SD**	**Total**	**Weight**	**IV, Random, 95% CI**
**1.1.1 Post-cesarean pain**								
Baber M 2015	25.4	14.4	34	23.6	12.2	64	33.1%	0.14 [-0.28, 0.55]
Stauble ME 2014	4.55	2.14	51	4.34	2.48	105	51.2%	0.09 [-0.25, 0.42]
VanderVaart S 2011	3.4	1.46	28	3.78	1.8	17	15.7%	-0.23 [-0.84, 0.37]
**Subtotal (95% CI)**			**113**			**186**	**100.0%**	**0.05 [-0.19, 0.29]**
Heterogeneity: Tau^2 = 0.00; Chi^2 = 1.07, df = 2 (P = 0.59); I^2 = 0%								
Test for overall effect: Z = 0.44 (P = 0.66)								
**Total (95% CI)**			**113**			**186**	**100.0%**	**0.05 [-0.19, 0.29]**
Heterogeneity: Tau^2 = 0.00; Chi^2 = 1.07, df = 2 (P = 0.59); I^2 = 0%								
Test for overall effect: Z = 0.44 (P = 0.66)								
Test for subgroup differences: Not applicable								

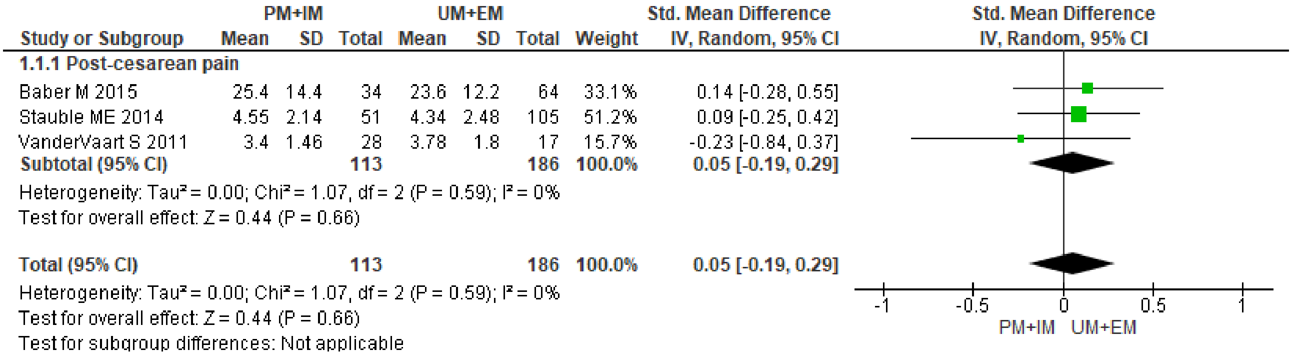

Supplementary Table 2. Quality assessment of studies included in the systematic review by the Q-Genie tool.

Author, year [Ref]	Question 1	Question 2	Question 3	Question 4	Question 5	Question 6	Question 7	Question 8	Question 9	Question 10	Question 11	Final score	Quality of the study
Landau R, 2008 [27]	7	7	7	5	4	5	6	7	5	3	7	63	Good
Sia AT, 2008 [18]	4	6	7	6	1	3	7	7	7	3	7	58	Good
Tan E, 2009 [19]	7	5	7	6	1	2	4	7	5	5	7	56	Good
Sia AT, 2010 [28]	7	6	7	7	1	2	7	5	5	3	7	57	Good
Tsai FF, 2010 [29]	7	7	7	7	4	5	6	7	4	3	5	62	Good
Wong CA, 2010 [21]	7	7	7	5	4	2	6	7	4	3	7	59	Good
De Capraris A, 2011 [30]	7	7	7	5	4	4	4	7	4	3	4	56	Good
VanderVaarrt S, 2011 [31]	7	6	7	5	3	7	1	6	7	3	5	57	Good
Camorcia M, 2012 [32]	7	7	6	5	4	2	2	5	4	3	4	49	Good
Landau R, 2013 [33]	7	7	6	4	4	7	5	7	5	3	7	62	Good
Boswell MV, 2013 [22]	7	5	5	4	1	3	2	5	5	3	7	47	Good
Ginosar Y, 2013 [34]	7	7	5	6	4	3	6	7	5	3	6	59	Good
Quinta R, 2014 [35]	7	5	5	6	1	1	2	5	2	3	3	40	Moderate
Stauble ME, 2014 [36]	7	5	7	3	1	2	2	7	7	3	5	49	Good
Baber M, 2015 [23]	7	4	7	5	1	3	1	7	6	5	5	51	Good
Xu GH, 2015 [37]	7	6	7	5	1	3	6	7	3	3	5	53	Good
Pettini E, 2018 [38]	7	5	6	3	1	2	1	2	3	3	2	35	Poor

Supplementary Table 2. Quality assessment of studies included in the systematic review by the Q-Genie tool (continued).

Author, year [Ref]	Question 1	Question 2	Question 3	Question 4	Question 5	Question 6	Question 7	Question 8	Question 9	Question 10	Question 11	Final score	Quality of the study
Xie W, 2018 [39]	7	6	7	7	4	2	7	5	7	3	6	61	Good
Lv J, 2018 [24]	7	4	5	7	1	5	2	6	6	3	4	50	Good
Zhang J, 2018 [20]	7	5	5	5	1	5	2	6	2	3	4	45	Moderate
Zgheib NK, 2018 [40]	7	7	7	7	3	5	6	7	2	3	5	59	Good
Kung CC, 2018 [41]	7	6	1	7	4	2	6	7	5	3	5	53	Good
Wang L, 2019 [42]	7	7	7	7	4	6	3	7	5	3	5	61	Good
Xiaohong Y, 2020 [43]	7	5	7	5	1	1	2	6	3	3	3	43	Moderate
Chen Y, 2023 [17]	7	5	7	5	1	4	6	7	5	3	7	57	Good
Shu X, 2024 [44]	7	7	7	7	4	4	4	6	2	3	7	58	Good
Li W, 2024 [45]	7	5	7	3	4	2	7	2	2	3	3	45	Moderate

Question 1. Please rate the study on the adequacy of the presented hypothesis and rationale. **Question 2.** Please rate the study on the classification of the outcome (e.g. disease status or quantitative trait). **Question 3.** Please rate the study on the description of comparison groups (e.g. cases and controls). **Question 4.** Please rate the study on the technical classification of the exposure (i.e. the genetic variant). **Question 5.** Please rate the study on the non-technical classification of the exposure (i.e. the genetic variant). **Question 6.** Please rate the study on the disclosure and discussion of sources of bias. **Question 7.** Please rate whether the study was adequately powered. **Question 8.** Please rate the study on description of planned analyses. **Question 9.** Please rate the study on the statistical methods. **Question 10.** Please rate the study on the description and test of all assumptions and inferences. **Question 11.** Please rate the study on whether conclusions drawn by the authors were supported by the results.
